# Adaptative survival of Aspergillus fumigatus to echinocandins arises from cell wall remodeling beyond β−1,3-glucan synthesis inhibition

**DOI:** 10.1038/s41467-024-50799-8

**Published:** 2024-07-31

**Authors:** Malitha C. Dickwella Widanage, Isha Gautam, Daipayan Sarkar, Frederic Mentink-Vigier, Josh V. Vermaas, Shi-You Ding, Andrew S. Lipton, Thierry Fontaine, Jean-Paul Latgé, Ping Wang, Tuo Wang

**Affiliations:** 1 Department of Chemistry, Michigan State University, East Lansing, MI USA; 2 MSU-DOE Plant Research Laboratory, East Lansing, MI USA; 3 National High Magnetic Field Laboratory, Tallahassee, FL USA; 4 Biochemistry and Molecular Biology, Michigan State University, East Lansing, MI USA; 5 Department of Plant Biology, Michigan State University, East Lansing, MI USA; 6 Environmental Molecular Sciences Laboratory, Pacific Northwest National Laboratory, Richland, WA USA; 7 Institut Pasteur, Université Paris Cité, INRAE, USC2019, Unité Biologie et Pathogénicité Fongiques, F-, 75015 Paris, France; 8 Institute of Molecular Biology and Biotechnology, University of Crete, Heraklion, Greece; 9 Departments of Microbiology, Immunology and Parasitology, Louisiana State University Health Sciences Center, New Orleans, LA USA; 10 Present Address: National High Magnetic Field Laboratory, Tallahassee, FL USA

**Keywords:** Solid-state NMR, Polysaccharides, Fungal biology, Antifungal agents, Antimicrobial resistance

## Abstract

Antifungal echinocandins inhibit the biosynthesis of β−1,3-glucan, a major and essential polysaccharide component of the fungal cell wall. However, the efficacy of echinocandins against the pathogen *Aspergillus fumigatus* is limited. Here, we use solid-state nuclear magnetic resonance (ssNMR) and other techniques to show that echinocandins induce dynamic changes in the assembly of mobile and rigid polymers within the *A. fumigatus* cell wall. The reduction of β−1,3-glucan induced by echinocandins is accompanied by a concurrent increase in levels of chitin, chitosan, and highly polymorphic α−1,3-glucans, whose physical association with chitin maintains cell wall integrity and modulates water permeability. The rearrangement of the macromolecular network is dynamic and controls the permeability and circulation of the drug throughout the cell wall. Thus, our results indicate that echinocandin treatment triggers compensatory rearrangements in the cell wall that may help *A. fumigatus* to tolerate the drugs’ antifungal effects.

## Abstract (web summary)

Echinocandins inhibit the biosynthesis of β−1,3-glucan, an essential component of the fungal cell wall, but their efficacy against the pathogen *Aspergillus fumigatus* is limited. Here, Dickwella Widanage et al. show that *A. fumigatus* responds to echinocandin treatment by remodelling various cell wall polymers, thus maintaining cell wall integrity and modulating the permeability and circulation of the drug in the cell wall.

## Introduction

**Fig. 1:**
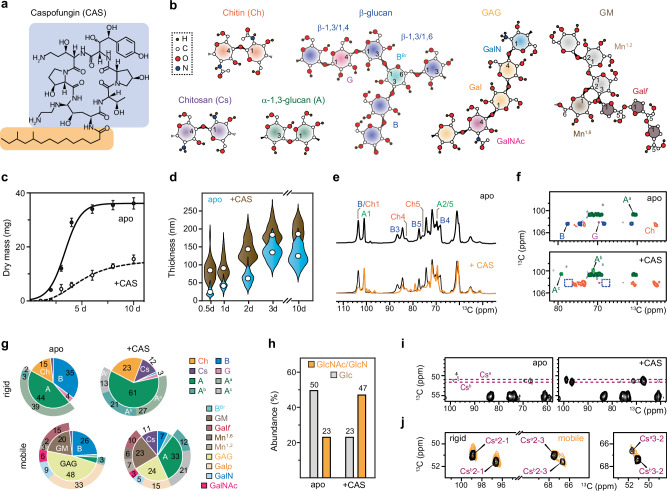
Alternation of cell wall polymer composition due to caspofungin treatment. **a** Chemical structure of caspofungin (CAS), highlighting the cyclic peptide in blue and the lipid component in yellow. **b** Simplified structures of fungal cell wall polysaccharides. NMR abbreviations are provided for each polysaccharide or monosaccharide unit. **c** Growth profiles of *A. fumigatus* without (apo) and with caspofungin as a change in dry mass. **d** Cell wall thickness of young and old samples determined using TEM images. Open circles: mean values. *n* = 75 for 0.5 d and 2 d apo samples. *n* = 120 for 0.5 d, 1 d, 10 d CAS-treated samples, and 2 d apo samples. *n* = 200 for other samples. **e** 1D ^13^C CP spectra of 3-day-old *A. fumigatus* samples showing the compositional difference of rigid polysaccharides. Top: apo sample; bottom: overlay of spectra from apo (black) and caspofungin-treated (orange) samples. **f** Comparison of 2D ^13^C-^13^C spectra of 3-day-old cell walls. The missing peaks of β−1,3-glucans in drug-treated samples are highlighted using blue boxes. **g** Molar composition of rigid (top) and mobile (bottom) cell wall polysaccharides of 3-day-old samples estimated using volumes of resolved cross peaks in 2D ^13^C CORD and DP refocused J-INADEQUATE spectra. Ch: chitin, Cs: chitosan, B: β−1,3-glucan, G: β−1,4-glucose residue, A: α−1,3-glucan. For α−1,3-glucan in the rigid portion, the inner pie chart displays the total amount, while the outer circle shows the individual content of three subtypes (A^a^, A^b^, and A^c^). B^Br^: β−1,3,6-glucose residue (the branching point), GM: galactomannan, Gal*f*: galactofuranose, Mn^1,2^: α−1,2-mannose, Mn^1,6^: α−1,6-mannose. For the mobile phase, the inner pie chart depicts the total content of each polysaccharide, while the outer circle shows the monosaccharide units or subtypes. **h** Changes of the total Glc (α- and β-glucan) and GlcN/GlcNAc (chitosan and chitin) in 3-day-old cell wall using GC-MS and HPLC after acid hydrolysis. **i** Detection of two chitosan types (Cs^a^ and Cs^b^) in drug-treated 3-day-old sample in 2D CORD spectra. **j** Presence of both chitosan forms in 3-day-old caspofungin-treated sample across both rigid fraction (black; CORD spectra) and mobile fraction (yellow; sheared refocused DP J-INADEQUATE spectra). For **c** and **d**, data are mean ± s.e. *n* = 3 replicates for **c** and *n* = 200 in 10 cells for each sample in **d**. Source data are provided as a Source Data file.

Invasive *Aspergillus* infections have high and increasing occurrence and mortality among immunocompromised and immunocompetent patients^1–3^, including, more recently, critically ill individuals suffering from SARS-CoV-2 infections^4,5^. There are currently a few antifungal agents available, with often therapeutic failures and a recently observed increase in drug resistance^6–9^. The cell wall is a prominent target for the selection of very specific antifungal drugs. Therefore, echinocandins, such as caspofungin and micafungin, have been approved for clinical applications^10–12^. These cyclic hexapeptides, which possess modifiable aliphatic tails (Fig. 1a), inhibit the biosynthesis of β-1,3-glucan, a major and essential structural polysaccharide in the fungal cell wall^10,13^.

As an effective fungicide for *Candida* species, echinocandins are being used as preferred agents for treating invasive candidiasis^14,15^. However, echinocandins only have relatively low efficacy in vitro and in vivo against *A. fumigatus*, the most ubiquitous airborne fungal pathogen responsible for pulmonary aspergillosis, and other filamentous fungi^16,17^. Echinocandins only exert a fungistatic effect on *A. fumigatus*, causing lysis to the hyphal tips without completely preventing growth^18^. Understanding the low efficacy of these β-1,3-glucan synthase inhibitors against *A. fumigatus* would benefit therapeutic application. Given the highly dynamic nature of the fungal cell wall, which undergoes continuous modifications in response to external stresses^19–22^, a plausible explanation could be the occurrence of specific compensatory mechanisms at the cell wall level promoting fungal survival by counteracting β-1,3-glucan reduction in filamentous fungi.

Recently, magic-angle spinning (MAS) ssNMR methods have unveiled the supramolecular arrangement of key polysaccharide components (Fig. 1b) within fungal cell wall architecture^23–25^. High-resolution data collected on intact *A. fumigatus* mycelia have demonstrated that α-1,3-glucan associates with chitin to form a robust mechanical scaffold, which is dispersed within a mobile and hydrated mesh formed by branched β-glucans, and enclosed by a dynamic outer shell rich in galactomannan (GM) and galactosaminogalactan (GAG)^26,27^. This nanoscale architecture was observed to undergo significant rearrangements in response to biosynthesis deficiencies in structural polysaccharides and during morphotype transitions throughout fungal life cycles^28,29^. Such methodology has been applied now to understand the structural modifications of *A. fumigatus* cell walls when this fungus is treated with echinocandins.

Here, ssNMR and dynamic nuclear polarization (DNP) techniques, in conjunction with transmission electron microscope (TEM) and atomic force microscopy (AFM) imaging, biochemical analysis, and molecular dynamics (MD) simulations, have unveiled major unexpected structural and dynamic changes in the mycelial cell wall of *A. fumigatus* treated by echinocandins. An echinocandin-induced augmentation of the chitin content is the first event associated with its partial deacetylation, leading to a significant amount of chitosan in the treated fungus. While only a low amount of β-glucan remains, it exhibits an atypical structure characterized by a high degree of branching. To compensate for the loss of most β-glucans, an increase in α-1,3-glucan content, along with the emergence of two novel types of semi-dynamic α-1,3-glucans, regenerates the soft matrix. The physical associations of chitosan, α-1,3-glucan, and chitin provide critical mechanical support, essential for preserving cell wall integrity. Moreover, the composition and structure of GAG and GM residing on the cell surface have been reshuffled. These compositional changes further lead to enhanced stiffness and hydrophobicity of the polymer network within the cell wall. These modifications induced by echinocandins have provided heretofore unavailable molecular insights into their limited effectiveness and defined structural paradigms for strategies to improve their efficacy.

## Results

### Caspofungin alters the carbohydrate core

Under our experimental condition, the addition of 2.5 µg/mL caspofungin resulted in a notable decrease in the growth of *A. fumigatus*, reaching only 25-50% of the growth observed in the untreated culture, with variation depending on the duration of growth (Fig. 1c). Caspofungin led to a 2–3-fold increase in cell wall thickness in younger cultures (0-2 days) and a 1.5-fold increase in older cultures (3-10 days), elevating the average thickness from 133 nm to 182 nm for 3-day-old samples (Fig. 1d and Supplementary Table 1). Uniformly ^13^C, ^15^N-labeled *A. fumigatus* mycelia were subjected to ssNMR analysis to identify molecular-level factors driving this microscopic-scale restructuring. Initial screening by one-dimensional (1D) ^13^C spectra confirmed the reduction of the β-1,3-glucan content in the rigid structural domain (Fig. 1e). The intensity reduction of characteristic peaks of β-1,3-glucan carbons (B3 at 86 ppm, B4 at 69 ppm, and B5 at 77 ppm), notably a 94% drop in the B3 peak, was observed in the echinocandin-treated sample. These changes were reproducibly observed across three sample batches (Supplementary Fig. 1).

Further two-dimensional (2D) ^13^C-^13^C/^15^N correlation spectra were acquired to resolve a large number of carbon sites in fungal glycans (Supplementary Fig. 2). As anticipated, the signals of β-1,3-glucans became almost negligible in the drug-treated cell walls (Fig. 1f). Analysis of peak volumes revealed that caspofungin decreased the β-1,3-glucan content in the rigid portion of the cell wall from 35% to 1% in 3-day-old *A. fumigatus* cultures, while the amount of chitin increased by 1.5-2-fold (Fig. 1g and Supplementary Table 2). Chemical analysis of the sugar composition through acid hydrolysis and HPLC validated the reduction in glucan content and the elevation of chitin level in the cell wall (Fig. 1h and Supplementary Table 3).

The content of chitosan, the deacetylated form of chitin, was initially minimal in the apo sample, but after caspofungin treatment, it increased to comprise 11-12% of the cell wall carbohydrates (Fig. 1i). Two distinct forms of chitosan molecules were identified, equally populated and evenly distributed across both the rigid and mobile fractions of the cell wall (Fig. 1j).

**Fig. 2:**
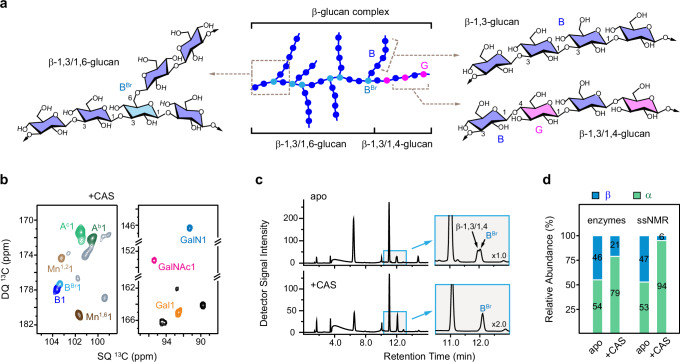
Caspofungin remodels the structures of β- and α-glucans. **a** Diagram illustrating the complex structure of β-glucans in *A. fumigatus* cell walls. NMR abbreviations are introduced to annotate different linkages in the main chains and branches. **b** Mobile polysaccharides detected using 2D ^13^C DP J-INADEQUATE spectrum resolving the β−1,3-linked (B) and β−1,3,6-linked (B^Br^) glucose units, as well as two novel forms of α−1,3-glucans (A^a^ and A^b^). **c** Qualitative analysis by HPLC shows the presence of β-glucan complex and the reduced amount of β-1,3/1,4-glucan domains after caspofungin treatment in 3-day-old cultures. **d** Percentages of α- and β-glucans in 3-day-old *A. fumigatus* samples determined by degrading the cell wall with recombinant α- and β-glucanases or using untreated cell walls by ssNMR.

The atomic resolution of ssNMR allowed us to discern intricate structural characteristics of β-glucan complex within intact *A. fumigatus* cell walls (Fig. 2a). This includes spectroscopic identification of glucopyranose residues bearing β-1,3, β-1,3,6, and β-1,4 linkages, with a particular focus on distinguishing the branching points (B^Br^) from the predominant linear chains (Fig. 2b and Supplementary Fig. 2c). The residual β-glucan surviving caspofungin treatment was hyperbranched through many β-1,3,6-linkages while its content of β-1,3/1,4-linked sequence was lower (Fig. 2c and Supplementary Fig. 3). These results show that the β-1,3/1,4-linked oligosaccharides are bound to the β-1,3 linear glucans targeted by caspofungin, while the branched β-1,3/1,6-glucan is part of the mobile fraction of the cell wall.

Caspofungin dramatically increased the amount of α-1,3-glucan, from 44% to 61% and from 4% to 33% in the rigid and mobile fractions, respectively (Fig. 1g and Supplementary Table 4). The accumulation of α-glucan compared to β-glucans was further validated using chemical assays (Fig. 2d and Supplementary Table 5). Apart from the major type of α-1,3-glucan (A^a^) primarily found in the rigid fraction, two other magnetically inequivalent forms (A^b^ and A^c^) were observed in both the rigid (Fig. 1f) and mobile fractions (Fig. 2b) of the caspofungin-treated sample. These two new types of α-1,3-glucans have not been reported previously and their chemical identities were confirmed by their absence from an *A. fumigatus* mutant and other fungal species that lack α-1,3-glucan (Supplementary Fig. 4). The three types of α-1,3-glucans exhibited distinct C3 chemical shift, ranging from 85 ppm for type-a to 79-81 ppm for types-b and c. Since C3 denotes the carbon position of the glycosidic linkage in this polysaccharide, the observed differences imply conformational alterations reflected in changes concerning the orientations of the glycosidic bonds and the adjacent sugar residues along the polymer. While the type-b and c forms exhibited very weak signals in the apo sample, they showed strong peaks in the *A. fumigatus* cell walls after caspofungin treatment (Supplementary Fig. 5). Thus, α-1,3-glucan simultaneously increased its amount and altered its structure to compensate for the loss of β-1,3-glucan. *A. fumigatus* cultures treated with anidulafungin and micafungin displayed spectra resembling those of caspofungin-treated cell walls (Supplementary Fig. 6), indicating similar structural changes induced by these echinocandins.

Other mobile polysaccharides, primarily GAG and GM, were largely conserved as shown by their comparable chemical shift fingerprints (Supplementary Fig. 7). Caspofungin halved the GAG content but did not perturb the molecular composition of the three sugar residues (Gal*p*, GalN, and GalNAc) within this heteroglycan (Fig. 1g and Supplementary Table 3). Due to the cationic state of GalN (present as GalNH_3_^+^) at cellular pH, the observed decrease in the GAG content will reduce the charge of the cell wall surface and affect the adhesive property. Although the total amount of galactomannan (GM) remained unchanged, the proportion of Gal*f* decreased by one-third (Fig. 1g), indicative of shorter or fewer galactofuran sidechains in caspofungin-treated mycelia. The carbohydrate-to-protein/lipid ratio remained stable following treatment with caspofungin (Supplementary Figs. 8, 9 and Table 6), as well as anidulafungin and micafungin (Supplementary Fig. 6d). This indicates that the overall quantity of structural polysaccharides remained effectively preserved after exposure to echinocandins. Fluctuations in lipid and protein content were observed during shorter incubation periods (Supplementary Fig. 10), likely originating from a combination of extracellular and intracellular sources, including plasma membranes (Supplementary Fig. 11).

**Fig. 3:**
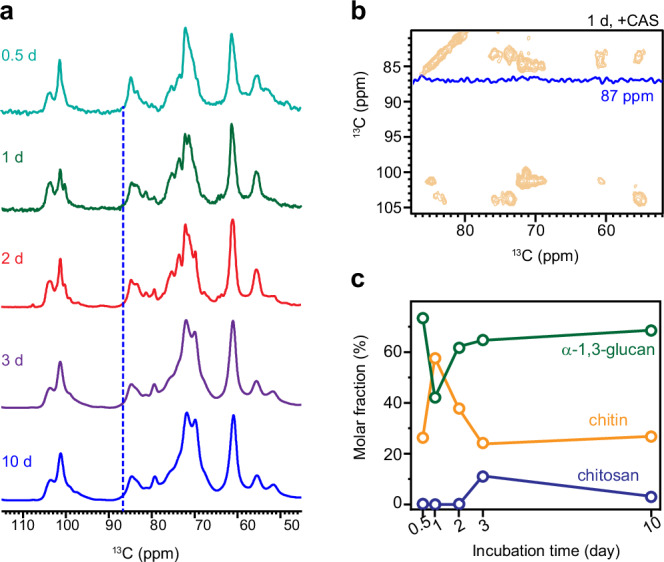
Evolution of polysaccharide composition during caspofungin treatment. **a** 1D CP spectra of caspofungin-treated *A. fumigatus* samples prepared with varied duration of culture. Blue dashed line indicates the characteristic position for the carbon-3 of β-1,3-glucan. **b** 2D ^13^C CORD spectra of one-day-old caspofungin-treated *A. fumigatus* confirming the absence of β-1,3-glucans. The 1D cross-section is extracted at 86.5 ppm, the characteristic chemical shift for carbon 3 of β-1,3-glucan. **c** Estimation of rigid polysaccharide fractions derived from spectral deconvolution.

The evolving biopolymer composition over time was assessed by analyzing spectra of younger cultures. No resolvable signal for β-glucan in the rigid phase could be detected in *A. fumigatus* samples cultured between 0.5–10 days (Fig. 3a, b). Spectral deconvolution and subsequent analysis of peak area have shown that chitin emerged as the predominant fraction, constituting 60% of intact cell wall domains after 1 day of caspofungin exposure, reinforcing the cell wall for survival (Fig. 3c and Supplementary Fig. 12). However, its proportion subsequently declined rapidly to one-fifth of the rigid molecules after 3 days, while its acetylated form, chitosan, concurrently increased to 10%. The fluctuations of chitin consistently opposed those of α-1,3-glucans, suggesting their shared roles in maintaining cell wall structures.

### Reduced water retention and polymer dynamics

**Fig. 4:**
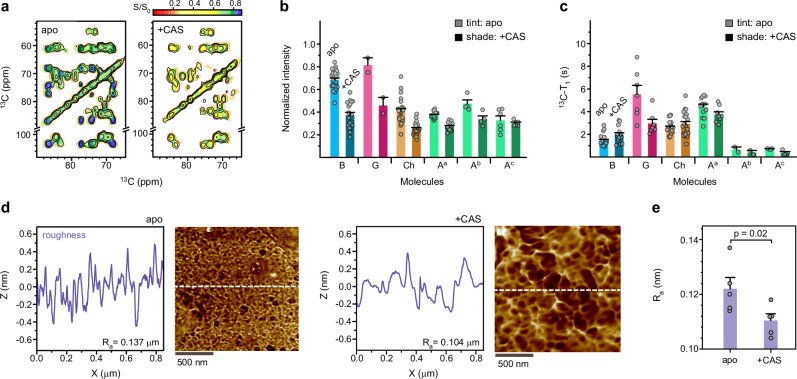
Effect of caspofungin on water accessibility, biopolymer rigidity, and surface roughness. **a** Hydration map of 3-day-old *A. fumigatus* without (top) and with (bottom) caspofungin treatment. This intensity map plotted the ratios (S/S_0_) of peak intensities from the water-edited spectrum (S) detecting hydrated molecules, relative to those from the control spectrum (S_0_) representing equilibrium conditions. **b** Relative intensities (S/S_0_) of different carbon sites indicating the extent of water association of cell wall polysaccharides. Data are shown for apo and drug-treated samples: β-1,3-glucan (B; *n* = 25, 19), chitin (Ch; *n* = 25, 25), β-1,4-glucose units (G; *n* = 3, 3), and three subtypes of α-1,3-glucan (A^a^, *n* = 16, 15; A^b^, *n* = 4, 4; A^c^, *n* = 6, 6). **c** ^13^C-T_1_ relaxation time constants for different carbon sites in 3-day-old *A. fumigatus* samples. Data are shown for carbohydrates in both apo and drug-treated samples: B (*n* = 20, 15), Ch (*n* = 20, 20), G (*n* = 7, 7), A^a^, (*n* = 12, 8), A^b^ (*n* = 2, 2), and A^c^ (*n* = 6, 6). Data in panels **b** and **c** are presented as mean ± s.e., with data points superimposed on the bar diagram. **d** Lateral surface roughness profiles and corresponding AFM images of 3-day-old *A. fumigatus* without (apo, left) and with (+CAS, right) caspofungin exposure, showing difference in the average roughness (R_a_). The white dashed lines denote the locations from which representative roughness profiles were derived. **e** Surface roughness determined by analyzing the lateral roughness profiles from five AFM images for both apo and drug-treated samples. Data are mean ± s.e. with *n* = 5 for apo and caspofungin-treated samples. The statistically significant differences (α = 0.02) were identified by unpaired Student *t*-test. Source data are provided as a Source Data file.

A key finding is that caspofungin treatment leads to a reduction in the water accessibility of *A. fumigatus* cell walls, which was marked by the decline of intensities in 2D hydration maps (Fig. 4a). Such intensities reflect the capabilities of polymers to retain water molecules around various carbon sites (Supplementary Table 7). Upon caspofungin treatment, the relative intensities of α-glucan, chitin, and β-1,3-glucan in cell walls of 3-day-old cultures dropped by 27%, 39%, and 46%, respectively (Fig. 4b). This decrease in water retention and increase in hydrophobicity should contribute to the reduction in cell wall permeability.

The dynamical heterogeneity of cell wall polysaccharides was examined using ^13^C spin-lattice (T_1_) relaxation measurements, carried out in a pseudo-3D format to enhance spectral resolution (Supplementary Fig. 13). Within each sample, β-1,3-glucans showed high mobility on the nanosecond timescale, with relatively short ^13^C-T_1_ time constants of 1–3 s (Fig. 4c and Supplementary Table 8). Chitin, the major form of α-1,3-glucan (A^a^), and β-1,4-linked glucose units exhibited longer ^13^C-T_1_ values of 3-8 s. The stiffness of chitin and α-1,3-glucan is justified by their close association at the mechanical center, but the long T_1_ values of β-1,4-linked glucose units, typically identified in the mix-linked β-1,3/1,4-glucan, are unexpected^26^. These β-1,3/1,4-glucans constitute minor segments that are occasionally covalently connected to the major β-1,3/1,6-glucan domains^30^. The data suggest that β-1,3/1,4-glucan is intrinsically inflexible, either achieved by maintaining physical separation from the bulk domain of mobile β-glucans or due to spatial constraints imposed by packing interactions with rigid molecules like chitin or α-glucan.

Caspofungin reshapes the dynamical landscape of the mechanical core in *A. fumigatus* cell walls. After treatment, the residual β-1,3-glucan (which transforms into highly branched β-1,3/1,6-glucan) was rigidified, with its average ^13^C-T_1_ increased apparently from 1.4-s to 2.0-s (Fig. 4c). Because most β-1,3-glucans residing in the soft matrix were eliminated due to drug inhibition, the remaining ones should primarily pack with rigid molecules. Caspofungin also rigidified chitin and increased its ^13^C-T_1_ from 2.6-s to 2.9-s. This could be attributed to the promoted aggregation of chitin chains in the absence of filling molecules (β-1,3-glucans). Conversely, β-1,4-linked glucose residues experienced an opposite shift, with almost halved ^13^C-T_1_ (from 5.5-s to 3.0-s). The removal of β-1,3-glucans has significantly impacted the stability of β-1,3/1,4-glucans, probably because these two polysaccharides were covalently linked to each other^30,31^. Similarly, the major form of α-1,3-glucan became moderately more dynamic, and the two minor forms are inherently dynamic, which should help to partially fulfill the diverse roles of the now-removed β-1,3-glucan. These two newly identified forms of α-1,3-glucans also displayed a distinct double-exponential feature (Supplementary Fig. 14 and Table 9), revealing their distribution across dynamically distinct regions.

While NMR uncovers dynamics and hydration at the molecular level, AFM provides direct measurement of cell wall surface topography and mechanical properties. Recent studies on *A. nidulans* have used AFM and subresolution imaging methods to determine the elastic modulus and its spatial gradient within the hyphae^32–34^. We analyzed the cellular-level surface roughness of 3-day-old *A. fumigatus* cultures using AFM (Fig. 4d) and observed a decrease in the lateral roughness in the drug-treated sample (Fig. 4e), indicating a smoothing effect of the fungal cell surface by caspofungin treatment.

### Rearrangement of the macromolecular network

**Fig. 5:**
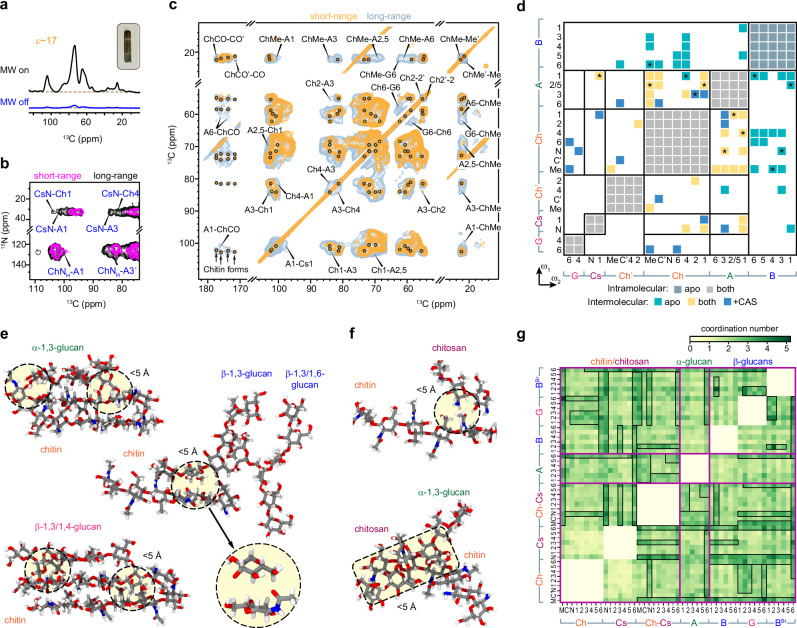
Intermolecular contacts in 3-day-old apo and caspofungin-treated *A. fumigatus.* **a** DNP enhances NMR sensitivity of the caspofungin-treated 3-day-old sample by 17-fold when microwave (MW) is activated. Inset shows the DNP sample with 30 mg hydrated mycelial material enclosed in a 3.2-mm sapphire rotor. Dash lines mark the baseline of the spectra. **b** DNP 2D ^15^N-^13^C correlation spectra of caspofungin-treated sample. Interactions happen between the ^15^N-site of the chitin amide (ChN_H_) or chitosan amine (CsN) and the carbons of polysaccharides. For example, CsN-A1 represents the cross peak between chitosan nitrogen with α−1,3-glucan carbon 1. **c**, DNP 2D ^13^C-^13^C correlation spectra of caspofungin-treated sample. Most interactions happen between α-glucan and chitin. The carbonyl region showed four types of chitin signals. **d** Site-specific summary of intermolecular cross peaks identified among different polysaccharides. Diagonal regions exhibit intramolecular cross peaks. Off-diagonal regions show intermolecular interactions happening only in the apo sample (green), only in the drug-treated sample (blue), or in both samples (yellow). Strong intermolecular interactions from short-mixing spectra are marked with asterisks. The plot can be asymmetric relative to the diagonal due to the directionality of polarization transfer, e.g., Ch1-A3 differs from A3-Ch1. The left and bottom axes indicate the carbohydrate carbon numbers observed in indirect (ω_1_) and direct (ω_2_) spectral dimensions. Representative short-range interactions observed during 1 *μ*s all-atom MD are shown between **e**, chitin, and glucans, and **f**, chitosan, and other polymers. Atoms in gray, red, blue, and white represent carbon, oxygen, nitrogen, and hydrogen, respectively. Packing interactions within 5 Å are highlighted. **g** Contact map of intermolecular interactions within 5 Å identified in MD models. The coordination number (see Methods) represents the number of contacts between two carbon sites of different polysaccharides. Magenta lines separate the sections of chitin/chitosan (Ch: chitin; Cs: chitosan; Ch-Cs: chitin-chitosan copolymer), α−1,3-glucan (A), and β-glucans (B: linear β−1,3-glucan; G: β−1,3/1,4-glucan; B^Br^: β−1,3/1,6-glucan). Carbon and nitrogen numbers are provided on both axes (M: Me or methyl; C’ carbonyl). Regions boxed in black highlight extensive interactions.

Fungal cell walls undergo significant spatial rearrangement in response to caspofungin. Assessment of the nanoscale assembly of macromolecules is enabled by the DNP technique, which relies on electron polarization to provide a 17-22-fold enhancement on the NMR sensitivity of two 3-day-old *A. fumigatus* samples (Fig. 5a)^35–37^. This enhancement results in a shortened experimental duration by 290 to 480 times. The cryogenic temperature required for DNP measurements has broadened the NMR linewidth and abolished the signals of highly disordered molecules such as GM and GAG, but the resolution remains adequate for resolving the diverse carbon and nitrogen sites of major polysaccharides and visualizing their sparsely populated packing interfaces (Supplementary Fig. 15).

Caspofungin-treated samples showed unambiguous cross-peaks between the nitrogen of chitosan amine group (CsN) and the carbons of chitin and α-glucan (Fig. 5b), providing clear evidence of the nanoscale coexistence of these biomolecules. Notably, the amide nitrogen of chitin correlates predominantly with the carbons of α-1,3-glucan, forming a physically associated complex. This structural concept is further supported by the numerous ^13^C-^13^C cross-peaks between these two polysaccharides (Fig. 5c). In addition, four major forms of N-acetylglucosamine (GlcNAc) units were identified. These units engage in hydrogen-bonding to create crystalline microfibrils^38^, and inter-form cross-peaks can be detected between the carbonyls (ChCO-CO’) and between the methyl groups (ChMe-Me’) of different conformers. These cross peaks represent physical contacts of two molecules occurring on the sub-nanometer length scale.

A statistical summary of 114 intermolecular cross peaks revealed the elimination of intermolecular contacts involving β-1,3-glucans, which is an expected outcome due to the depletion of this polysaccharide by caspofungin (Fig. 5d and Supplementary Fig. 16). In contrast, drug treatment promoted the extensive associations between chitin and α-1,3-glucan, thereby stabilizing the polymer complexes formed by these two macromolecules. The elevated levels of chitin and α-1,3-glucan in the caspofungin-treated sample may have additionally played a role in facilitating the detection of their physical contact. Chitosan was found to be anchored to this chitin-α-1,3-glucan core, and caspofungin treatment has strengthened this packing interaction. Chitosan could potentially exist as a constituent of chitin-chitosan copolymers that are sometimes referred to as partially deacetylated chitin, or as fully deacetylated chains that are physically deposited onto the surface of chitin crystallites. Interestingly, β-1,4-linked glucose residues also showed several cross peaks with chitin. This suggests that β-1,3/1,4-glucan is trapped by chitin microfibrils, which explains the distinctive rigidity of this linear polymer as shown in Fig. 4c. Therefore, in the absence of the bridging molecule β-1,3-glucan, the stability of the *A. fumigatus* cell wall is sustained through many inter-allomorph interactions within chitin crystallites and intermolecular associations involving chitin, α-1,3-glucans, chitosan, and β-1,3/1,4-glucan, collectively ensuring the maintenance of structural integrity.

Despite the potential involvement of protein and lipid in the cell wall architecture, no observable interactions were detected between these non-carbohydrate components and the cell wall polysaccharides (Supplementary Fig. 17). This is likely due to the restricted physical interactions between these polymers. Alternatively, the disorder nature of protein-carbohydrate complexes may result in signal broadening and rendering them undetectable at the cryogenic temperature of DNP. Nonetheless, some proteins and lipids still exhibit observable signals under these conditions. Recent chemical and NMR analyses have reported covalent connections between structural proteins and GM/GAG through unusual alkali-resistant linkers that incorporate hydrophobic amino acid residues such as valines^29^. This structural pattern remains unaffected in the caspofungin-treated cell wall, as evident from the consistent signals of valine and other amino acids in the rigid phase of the *A. fumigatus* samples. It can be inferred that lipids and proteins have limited contact with cell wall polysaccharides, primarily associated with GM and GAG in the surface layer.

Microsecond-long all-atom molecular dynamics (MD) simulations have been employed to quantify intermolecular interactions and visualize polysaccharide packing interfaces in cell walls^39,40^. The solvated molecular system includes 14 polysaccharides, with two copies of each linear polysaccharides (chitin, chitosan, chitin-chitosan copolymer, α-1,3-glucan, β-1,3-glucan, and β-1,3/1,4-glucan) and the branched polymer β-1,3/1,6-glucan (Supplementary Fig. 18 and Movie 1). From microsecond-long unbiased MD trajectory, we systematically scanned over different cutoff distances to locate the best-fit trajectory, considering both short-range interactions (<7 Å) and long-range interactions (7-10 Å) based on the experimental observations provided by ssNMR. Chitin is physically aggregated with both α-1,3-glucan and the linear terminal chain of β-1,3/1,4-glucan (Fig. 5e and Supplementary Movie 2). The intermolecular distances are typically within 5.0 Å in the molecular trajectories, which rationalizes the strong cross-peaks observed among these polysaccharides in caspofungin-treated cell walls. Chitin also exhibited close-range interactions with both linear β-1,3-glucan and branched β-1,3/1,6-glucan; however, these molecules tend to be dispersed in space (Fig. 5e and Supplementary Movie 3). Chitin’s association with chitosan was stable, with nitrogen-carbon/nitrogen distances of less than 5.0 Å (Fig. 5f and Supplementary Movie 4), highlighting the importance of hydrogen bonds involving nitrogen sites in chitin amide and chitosan amine. Short-range interactions were also observed between chitin-chitosan copolymers and α-1,3-glucans (Fig. 5f). Two chitin polymers also tend to exist as a stacked assembly (Supplementary Movie 5), which can serve as the platform to accommodate external molecules, such as α-1,3-glucan and β-1,3/1,4-glucan as shown in Fig. 5e. These simulation results explained the origin of NMR cross peaks happening between the chitosan introduced by caspofungin treatment and the stiff scaffold of chitin and α-glucan in the cell wall.

The interatomic contact map (Fig. 5g) allows us to visualize extensive short-range interactions (within 5 Å) between the chitin methyl group and the pyranose rings of both α-1,3- and β-glucans. The amine nitrogen of chitosan also played a major role in interacting with all three types of β-glucans. α-1,3-glucan is capable of interacting with both chitin and chitosan, utilizing its carbon-6 that extends outside the pyranose ring. Alternatively, all carbon sites of α-1,3-glucan predominantly interacted with the chitin-chitosan copolymer, a component that is possibly present only in the caspofungin-treated *A. fumigatus* cells. Therefore, the NMR-observed strong cross-peaks between α-1,3-glucan and chitin/chitosan after the depletion of β-1,3-glucan by caspofungin were not surprising. These unbiased all-atom MD models provided detailed structural insights into the pairwise interactions between different functional groups present in fungal polysaccharides.

## Discussion

**Fig. 6:**
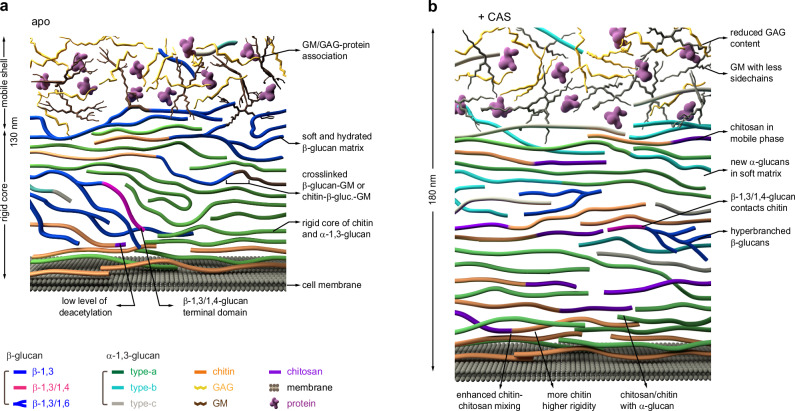
Schematic illustration of cell wall polysaccharide reorganization induced by caspofungin treatment. The illustration integrates NMR observations with the biochemical understanding of *A. fumigatus* cell walls. **a** Organization of cell wall polysaccharides in untreated 3-day-old *A. fumigatus* mycelial cell walls. The eight molecules shown in the figure include chitin (orange), α−1,3-glucan (green for type-a, cyan for type-b, and grey for type-c), β−1,3-glucan (blue, linear), β−1,3/1,6-glucan (blue, branched), β−1,3/1,4-glucan (magenta), chitosan (purple strands), GAG (yellow), GM (brown), cell membranes (dark grey), and proteins (purple particles). **b** Following caspofungin treatment, the amount of β−1,3-glucan is substantially reduced while the content of chitin, chitosan, and the two minor forms of α−1,3-glucans increased. The physical contacts among the remaining biopolymers also increased. These illustrative figures are constructed based on previous models^41,104^ built on biochemical and imaging studies, and with new insights from current NMR analysis regarding molecular composition and intermolecular contacts. The sizes and orientations of the polymers depicted within the cell wall are hypothetical.

The fungal cell wall is an elastic entity that withstands hydrostatic turgor pressure, with microfibrillar components restricting stretching and matrix components countering compression^41^. Most polymers are interconnected by covalent linkages and held together by hydrogen bonds rather than as separate components^42^. Previous chemical and imaging studies and recent NMR data showed that the *A. fumigatus* mycelial cell wall comprises a mobile domain primarily composed of GAG, GM, and associated proteins, and an inner layer consisting of a β-glucan network that encompasses the hydrophobic and rigid junctions formed by physically packed chitin and α-glucans (Fig. 6a)^27,29^. At the same time, β-1,3-glucan also covalently crosslinks chitin and GM together, as revealed by chemical analysis^43^. Our findings have further reshaped our perception of the response of fungal cell walls to echinocandins (Fig. 6b).

First, a prominent alteration observed was the modification of the two amino-sugar polymers. The elevated level of chitin, which is a classical compensatory response for *A. fumigatus* to caspofungin and other cell wall stresses^44,45^, was also shown in this study. It explains why chitin synthase mutants are more sensitive to caspofungin^46,47^ and why combining Nikkomycin Z, a chitin synthase inhibitor, with caspofungin synergistically enhances the efficacy of caspofungin^48,49^. The enhanced chitin biosynthesis reinforces the rigidity of the cell wall while maintaining the polymorphic nature of the chitin structure and its association with other forms. However, an apparition of the high amount of chitosan was unexpected. The septuple chitin deacetylase mutant of *A. fumigatus* is not more sensitive to caspofungin^50^, which would suggest that the chitosan likely does not play a significant role in maintaining the cell wall structure. Yet, recent data from *Aspergillus* species is illuminating the connection of chitosan with external stressors, including hypersaline conditions, as reported recently^51^, and caspofungin exposure, as demonstrated here.

Second, the unexpected emergence of two novel allomorphs of α-1,3-glucans exhibiting a wide distribution within both rigid and mobile phases was noteworthy. While similar conformational changes have been documented in plant polysaccharides like xylan and homogalacturonan due to interactions with other polysaccharides or divalent cations^52–54^, such phenomena have not been observed before in fungi. The stability of drug-treated cell walls is upheld by numerous physical interactions involving different chitin allomorphs and the interactions between chitin and α-1,3-glucans, which constrain molecular movements and restrict water accessibility within the cell wall. The increased resistance of the triple α-1,3-glucan synthase mutant to caspofungin compared to its parental strain^55^ also suggests that the modification of α-1,3-glucan by caspofungin not only plays a structural role in cell wall maintenance but also regulates caspofungin penetration into fungal cells. This is further corroborated by previous studies which have frequently observed α-1,3-glucan to be predominantly located externally in *A. fumigatus*^56,57^.

Third, under the experimental conditions tested which did not remove the entire cell wall β-glucan, the rigid and linear β-1,3-glucan almost vanishes, resulting in the subsequent removal of the covalently linked polysaccharide cores, such as (chitin-)β-1,3-glucan-GM and β-1,3-glucan-β-1,3/1,4-glucan, which are commonly found in untreated cells^43^. The remaining β-glucan predominantly exists within the mobile matrix and has undergone restructuring characterized by elevated β-1,6-branching, indicating that the extracellular remodeling process, controlled by members of the glycoside hydrolase GH-72 family^58^, remained unaltered. The β-1,3/1,4-glucan linear domains became physically associated with chitin. Interestingly, the *tft1*Δ mutant lacking β-1,3/1,4-glucan is slightly more sensitive to caspofungin^31^.

Fourth, a decrease in exopolysaccharide GAG content was noticed in caspofungin-treated cell walls, resulting in nearly equal proportions of GM and GAG on the cell wall surface. However, the impact of GAG on caspofungin susceptibility has been shown in the past to be minimum since mutants entirely lacking GAG or with increased GAG levels showed caspofungin susceptibility comparable to their parental strain^59,60^. GM also exhibits reduced content of Gal*f* sidechains in mycelia treated with the drug. Analysis of these galactose-based polysaccharides revealed altered galactose metabolism, likely as a compensatory mechanism for supporting cell wall integrity.

As β-glucan has been recognized as a key polysaccharide involved in covalently linking various components^61^, the success of the cell walls devoid of most β-1,3-glucans holds two significant implications. First, it suggests that other polysaccharides can substitute for the structural role of β-1,3-glucan, enabling the survival of those fungi with highly intricate cell walls. Second, it emphasizes the importance of packing interactions and physical properties in stabilizing the biopolymer assembly and supporting the cell wall’s function. The compromised water retention in the treated caspofungin due to the loss of β-glucan is key to maintaining water association within the cell wall. Third, the continuous and dynamic alterations in the concentration and composition of cell wall polymers induced by caspofungin, as shown by ssNMR data, are more rapid and complex than anticipated. Finally, these analyses also showed the importance of cell wall rigidity, and permeability in the drug influx or efflux.

Mutations in the *fks1* genes preventing the binding of echinocandins to the catalytic subunit FKS1 of β-1,3-glucan synthase^62,63^ have been identified as responsible for the drug’s limited efficacy. However, the survival of *A. fumigatus* during caspofungin treatment is largely FKS1-independent and arises from its tolerance to the drug, influenced by complex cellular and genetic interactions^64–66^. This tolerance includes well-documented phenomena such as the paradoxical effect, involving stress response pathways like Hsp90, calcineurin, and MAP kinases, and several transcription factors^64,65^. Twenty-one genes have been implicated in contributing to paradoxical growth^67^. Induction of reactive oxygen species (ROS) can also promote antifungal drug tolerance and the evolution of drug resistance^62^. Remodeling of the cell wall structure by drugs associated with the heterogeneity of fungal cell biology within a colony also significantly influences the subsequent penetration of cell wall-targeted drugs and the tolerance to these drugs^68^.

Another important aspect to be emphasized in future antifungal studies is the modification of the cell wall permeability to the drug since remodeled cell wall structure can hinder the subsequent penetration of cell wall-targeted drugs^68^. While it is anticipated that cell wall modifications will vary depending on the drug utilized, the current findings have advanced our comprehension of cell wall structural integrity and have suggested promising avenues for improving antifungal therapies using cell-wall-targeting drugs. New drug combinations may emerge utilizing rezafungin^69^, a new echinocandin, manogepix, which targets GPI-anchored proteins controlling native polysaccharide modifications^70^, and nanobodies^71^ targeting chitin synthase, as no efficient anti-chitin synthase drug besides nikkomycin, which has not been used clinically, have been identified to date. This study has shown that the biophysical methodology holds the potential for thoroughly elucidating the restructuring impacts of diverse antifungal compounds, including both cell-wall targeting agents and those that do not specifically focus on polysaccharides, on cell wall structures during the adaptive survival of human fungal pathogens.

## Methods

### Preparation of ^13^C, ^15^N-fungal materials

*A. fumigatus* (strain RL 578) was grown in Yeast Extract Peptone Dextrose (YPD) for seven days at 30 °C. Liquid cultures were prepared using a modified minimum medium containing 10.0 g/L of ^13^C-glucose and 6.0 g/L of ^15^N-sodium nitrate for uniformly labeling the fungal material^72^. The medium was adjusted to pH 6.5. Two batches of *A. fumigatus* cultures were prepared in parallel, and the mycelium was recovered after 3 days of growth at a time when both apo and treated mycelia were in a linear phase of growth, thus excluding cell wall modifications attributed to autolysis during the stationary or declining phase of the fungus. The concentration of caspofungin drug used was 2.5 µg/mL, estimated as MIC 50 (inhibiting 50 % of the mycelial growth). *A. fumigatus* was grown in 100 mL liquid medium in 250 mL Erlenmeyer flasks in a shaking incubator (210 rpm) at 30 °C. The mycelia were collected by centrifugation at 7000 g for 20 min. The harvested pellets were then washed thoroughly using 10 mM phosphate buffer (PBS, pH 7.4). For each sample, approximately 100 mg of the whole-cell material was packed into a 4-mm magic-angle spinning (MAS) rotor for ssNMR characterization. Another 30 mg of material was packed into a 3.2-mm sapphire rotor for DNP experiments. The native hydration level of the fungal cell was fully retained. Four additional samples were prepared under identical conditions and caspofungin concentration, with variations in culture duration (0.5 d, 1 d, 2 d, and 10 d) to understand the changes in polysaccharide composition. Additionally, two more batches of samples were prepared using the same protocol for 3-day-old cultures, but this time treated with micafungin and anidulafungin, each at a concentration of 2.5 µg/mL. In addition, *A. sydowii* was grown in minimum media with ^15^N-ammonium sulfate for 7 days at 28 °C, and *C. albicans* was grown in yeast nitrogen-based (YNB) without amino acids and ammonium sulfate with ^15^N-ammonium sulfate for 3 days at 30 °C. ^13^C-glucose was used for both cultures as the carbon source.

### TEM imaging

The harvested *A. fumigatus* mycelial cultures were fixed with 2.5% (vol/vol) glutaraldehyde and 2% (wt/vol) paraformaldehyde in 0.1 M PBS buffer (pH 7.4). The suspensions were centrifuged and embedded in 3% agarose gel. Subsequently, the samples were rinsed with 0.1 M PBS (pH 7.4), 0.05 M glycine, and then post-fixed with 2% osmium tetroxide (OsO_4_). The samples were rinsed thrice using deionized water. Acetone (50%, 70%, 80%, 90%, and 100%) and propylene oxide were used to dehydrate the samples in two 15 minutes cycles. Finally, a series of propylene oxide: Epon was used for infiltration. Ultrathin sections of the resulting samples were taken for TEM assessments. To increase the contrast during imaging, 1% uranyl acetate *En Bloc* staining was used. In total, ten samples were analyzed, comprising both untreated and caspofungin-treated *A. fumigatus* samples collected at five different culture durations of 0.5 d, 1 d, 2 d, 3 d, and 10 d. All images were taken on perpendicular cross-sections of hyphae using a JEOL JEM-1400 electron microscope.

### AFM analysis

Three-day-old untreated and caspofungin-treated *A. fumigatus* were used for AFM imaging in air. A single layer of cells was subjected on a glass slide and dried at room temperature. We employed the Bruker FastScan AFM system using ScanAsyst^TM^ imaging mode and SCANASYST-AIR AFM probes (Bruker). All AFM images were taken under the same imaging settings: scan size 1 μm, samples/line 512, scan rate 1 Hz, with a nominal spring constant of 0.4 N/m. For each sample, imaging was performed with at least three independently prepared samples. The average roughness (Ra) of the lateral surface profile was determined from five representative images for each sample using Gwyddion software and a *t*-test assuming unequal variance was performed.

### Cell wall fractionation and carbohydrate analysis

For carbohydrate analysis, mycelia were disrupted with 1 mm glass beads in 0.2 M Tris-HCl buffer, pH 8.0 with a fast-prep cell disruptor (MP-Bio). Crude cell wall fraction was collected by centrifugation (4500 × *g*, 10 min), washed three times with distilled water, and then fractionated into alkali-insoluble and alkali-soluble portions^30,73^. Hexose and hexosamine were quantified by colorimetric and chromatographic assays^30,73^. Neutral monosaccharides were analyzed by gas-liquid chromatography (GC) as alditol acetates obtained after hydrolysis (4 N trifluoroacetic acid, 100 °C, 4 h). Derivatized monosaccharides were separated and quantified on a DB5 capillary column (25 m x 0.32 mm, SGE) using a Perichrom GC apparatus (carrier gas, 0.7 bar helium; temperature program, 120-180 °C at 2 °C/min and 180–240 °C at 4 °C/min). To quantify the content of hexosamine, cell wall fractions were hydrolyzed with 6 N HCl at 100 °C for 6 h and were analyzed by high-performance anion exchange chromatography (HPAEC) with a pulsed electrochemical detector and an anion exchange column (CarboPAC PA-1, 4.6 × 250 mm, Dionex) using 18 mM NaOH as mobile phase at a flow rate of 1 mL/min; glucosamine and galactosamine were used as standards.

To quantify β-1,3-glucan and α-1,3-glucan in the cell wall, the alkali-insoluble fraction and the water-soluble supernatant fraction were submitted to enzymatic digestions. Fractions (1 mg/mL) were incubated with recombinant β-1,3-glucanase (LamA from *Thermotoga neapolitana*) and the mutanase from *Trichoderma harzianum*, respectively, in 20 mM sodium acetate buffer (pH 5.5) at 37 °C for 24 h. The reducing sugar released after enzyme digestion were quantified by the 4-hydroxybenzhydrazide (PABA) assay. To quantify β-1,6-branching in β-1,3-glucan and β-1,3/1,4-glucan, the enzyme digests of different cell wall fractions were subjected to HPAEC using a CarboPAC PA-1 column (4.6 × 250 mm, Dionex) at a flow rate of 1 mL/min; eluent A was 50 mM NaOH, and eluent B was 0.5 M NaOAc in 50 mM NaOH. The elution gradient was: 0–2 min, isocratic 98% A:2% B; 2–15 min, linear gradient from 98% A:2% B to 65% A:35% B; 15–35 min, linear gradient from 65% A:35% B to 30% A:70% B, followed by 100% B for 3 min. Glycosidic linkages were investigated by methylation of cell wall fractions followed by GC-MS analysis^73,74^. The amount of cell wall galactomannan was estimated from the mannose and galactose content of the alkali-insoluble fraction.

### SsNMR experiments for structural analysis

Most NMR experiments were conducted on a Bruker Avance 400 MHz (9.4 Tesla) spectrometer and an 800 MHz (18.8 Tesla) using 4-mm and 3.2-mm MAS HCN probes, respectively. Micafungin and anidulafungin-treated samples were analyzed using an 850 MHz NMR spectrometer. All experimental data, except those for MAS-DNP, were collected under 10–13.5 kHz MAS at 290 K. ^13^C chemical shifts were externally referenced to the adamantane CH_2_ peak at 38.48 ppm on the tetramethylsilane (TMS) scale. ^15^N chemical shifts were referenced to the liquid ammonia scale either externally through the methionine amide resonance (127.88 ppm) of the model tri-peptide N-formyl-Met-Leu-Phe-OH or using the ratio of the gyromagnetic ratios of ^15^N and ^13^C^75^. Typical radiofrequency field strengths, unless specifically mentioned, were 80–100 kHz for ^1^H decoupling, 62.5 kHz for ^1^H hard pulses, 50–62.5 kHz for ^13^C, and 41 kHz for ^15^N. The experimental conditions were listed in Supplementary Table 10.

One-dimensional (1D) ^13^C spectra were obtained using different polarization methods to selectively detect the rigid and mobile components of the fungal molecules. The rigid components were detected by 1D ^13^C cross-polarization (CP) using 1-ms contact time. Quantitative detection and mobile components located inside the cell wall were measured by 1D ^13^C direct polarization (DP) using short (2-s) and long (35-s) recycle delays, respectively. Spectral deconvolution was conducted on 1D ^13^C CP spectra of caspofungin-treated *A. fumigatus* samples cultured for 0.5, 1, 2, 3, and 10 days. The parameter provided by the DMfit software^76^ (version dmfit#20230120) is provided in Supplementary Table 11. 2D ^13^C-^13^C 53-ms CORD homonuclear correlation spectra were obtained to detect intramolecular cross-peaks and 2D ^15^N-^13^C N(CA)CX heteronuclear correlation spectra were obtained to detect amide signals of chitin^77^. To measure N(CA)CX, 0.6-ms ^1^H-^15^N CP, 5-ms ^15^N-^13^C CP contact times, and 100-ms DARR mixing time were used. The 2D DP refocused J-INADEQUATE spectra were obtained to detect mobile components with through bond connectivity. The assigned ^13^C and ^15^N signals were documented in Supplementary Table 12.

To determine the water accessibility of polysaccharides, water-edited 2D ^13^C-^13^C correlation spectra were obtained^78,79^. The experiment was initiated with ^1^H excitation followed by a ^1^H-T_2_ filter of 1.2-ms × 2, which eliminated 97% of the polysaccharide signals but retained 80% of water magnetization. The water magnetization was then transferred to the polysaccharide using a 4-ms ^1^H mixing period and then transferred to ^13^C through a 1-ms ^1^H-^13^C CP for site-specific detection. A 50-ms DARR mixing period was used for both the water-edited spectrum and a control 2D spectrum showing the full intensity. The relative intensity ratio between the water-edited spectrum and the control spectrum was quantified for all cell walls to reflect the relative extent of hydration (Supplementary Table 7). The intensities were pre-normalized by the number of scans of each spectrum. Hydration maps were generated using a python script integrated with nmrglue and matplotlib packages^80,81^.

Polysaccharide dynamics were probed using ^13^C spin-lattice (T_1_) relaxation, which was examined using a series of 2D ^13^C-^13^C correlation spectra with different z-filter durations (0-s, 0.1–s, 1-s, 3-s, and 9-s)^82^. The absolute intensity of each peak was quantified and then normalized by the number of scans. Relaxation data were fit using a single exponential function to obtain ^13^C-T_1_ time constants (Supplementary Table 8). Some relaxation curves exhibited a distinct double-exponential feature, particularly for the two new types of α-1,3-glucans, and therefore were fit separately using double exponential functions (Supplementary Table 9).

### MAS-DNP sample preparation and experiments

The stock solution containing 10 mM AMUPol was freshly prepared using a d_8_-glycerol/D_2_O/H_2_O (60/30/10 Vol%) solvent mixture, typically named DNP-matrix^83^. Around 30 mg of ^13^C,^15^N-labeled mycelial cells were mixed with 50 µL of stock solution and gently ground using a mortar and pestle for 10-15 min, which allows radicals to penetrate the porous cell walls. The material was then transferred to a 3.2-mm sapphire rotor for the DNP measurement. All DNP experiments were performed on a 600 MHz/395 GHz 89 mm-bore MAS-DNP spectrometer equipped with a gyrotron microwave source^84^. All spectra were measured using a 3.2-mm HCN probe under 8 kHz MAS frequency at 92 K. The microwave power was set to 12 W at the probe base. The enhancement factor of NMR sensitivity with and without microwave irradiation (ε_on/off_) was 17–32 for these samples. The buildup time varied between 5.2–13-s.

2D ^13^C-^13^C 100-ms PDSD were measured to detect intra-molecular correlations, followed by 20-ms PAR measurement to detect both intra- and inter-molecular correlations in carbohydrates of each cell wall^85^. The ^13^C and ^1^H irradiation frequencies were 56 kHz and 53 kHz during the PAR mixing. 2D ^15^N-^13^C N(CA)CX spectra were measured using the double-CP sequence^86^, with 1.0 ms contact time for the ^1^H-^15^N CP and 4.0 ms for the ^15^N-^13^C CP. In these ^15^N-^13^C experiments, ^13^C-^13^C mixing was achieved using a PDSD period of either 0.1 s for detecting intramolecular cross peaks or 3.0 s for detecting both intra- and inter-molecular cross peaks. The resonance assignment under DNP condition was presented in Supplementary Fig. 15a–d, with the chemical shifts documented in Supplementary Table 13. The identified long-range intermolecular cross peaks were listed in Supplementary Table 14. Spectral asymmetry was noted for some cross peaks, attributed to differing protonation states and relaxation properties of the two carbon sites engaged in intermolecular correlation (Supplementary Fig. 19), a phenomenon previously noted in cell wall systems^87,88^.

### Construction of atomic models for MD simulation

We first assembled model α- and β-glucans using CHARMM-GUI Glycan Modeler^89,90^. The degree of polymerization (D.P.) of each polysaccharide was set to six monomers, which allowed us to keep the total system size of the whole system under 200,000 atoms as detailed later. Although the D.P. of the polysaccharide in the native fungal cell wall is significantly higher, the current atomistic model allows us to evaluate the structure-based polymer interactions. The CHARMM-GUI Glycan Modeler then assembled the short linear polysaccharides, α-1,3-glucan, β-1,3-glucan, and β-1,3/1,4-glucan as well as the branched β-1,3/1,6-glucan (Supplementary Fig. 18a). Similarly, short chitin polymers were generated using the plugin chitin_builder^91^, within visual molecular dynamics (VMD 1.9.4a55)^92^. Chitin and chitosan are copolymers of 2-acetamido-2-deoxy-D-glucose (N-acetyl glucosamine, GlcNAc), and 2-amino-2-deoxy-D-glucose (glucosamine, GlcNH_2_), connected by β-1,4 glycoside linkages^93^. Chitin and chitosan might exist as a copolymer, with a variable degree of N-acetylation. In addition to the copolymer in our molecular system, we created separate polymers for chitin and chitosan to accurately track the individual interactions specific to each of the two polysaccharides. Since glucosamine in the existing CHARMM carbohydrate force field carries a charge^94,95^, we introduced a patch to modify the topology and convert glucosamine into a monosaccharide unit of chitosan by deleting hydrogen and reparametrizing the charges. The final system has two copies of each polysaccharide, which were solvated in a box of water using the solvate plugin in VMD 1.9.4a55, with 5 mM of NaCl added to the hydrated neutralized model using the autoionize plugin to VMD 1.9.4a55^92^. The atomistic model consists of 120,972 atoms after adding solvent and ions (Supplementary Fig. 18b).

### MD simulation and analysis of molecular trajectories

All-atom classical MD simulations for the models illustrated in Fig. 5e, f and Supplementary Fig. 18 were initially minimized using NAMD 2.14, to prepare for production simulations using the GPU resident integrator in NAMD 3.0a9. The simulation employed the CHARMM36 force fields for computing the interactions between carbohydrates^94,95^, water^96^, and ions^97^. Following CHARMM36 force field, we used a 12 Å cutoff and a force-switching function after 10 Å. Long-range electrostatics are treated using the particle mesh Ewald with a 1.2 Å grid spacing^98^. To enable a 2 fs timestep between force evaluations, covalent bonds involving hydrogen atoms were treated using the RATTLE algorithm^99^. The Langevin thermostat was set to maintain a temperature of 300 K using a 5 ps^−1^ damping coefficient^100^. A microsecond-long MD simulation was performed for an NPT ensemble, using a Langevin isotropic barostat that was set to maintain 1 atm pressure^101^.

The simulation trajectory was analyzed using VMD 1.9.4a55, which was enabled with Python 3.10.12 and Numpy 1.24.2^102^. In-house scripts were prepared in Tcl 8.6 and Python 3.10.12 programming software to calculate and quantify the intermolecular interactions between polysaccharides using the coordination number collective variable^103^. The coordination number (C_ij_) measures the pairwise contact between two atoms within a given polymer chain. This was implemented using a switching function defined as ${C}_{{ij}}={\sum }_{i}{\sum }_{j\ne i}1-{(|{{{{\boldsymbol{x}}}}}_{{{{\boldsymbol{i}}}}}-{{{{\boldsymbol{x}}}}}_{{{{\boldsymbol{j}}}}}|/{d}_{o})}^{n}/1-{(|{{{{\boldsymbol{x}}}}}_{{{{\boldsymbol{i}}}}}-{{{{\boldsymbol{x}}}}}_{{{{\boldsymbol{j}}}}}|/{d}_{o})}^{m}$. Short- and long-range interactions were defined using the cutoff parameter, which used the switching distance to define an interatomic contact, for all d « d_0_. Exponents n and m are set to 10 and 120, to steepen the switching profile. The equation for the coordination number is illustrated in Supplementary Fig. 18d for short-range and long-range cutoff 5 and 7 Å with the exponents *n* = 10 and m = 120. A contact map illustrating the polysaccharide interactions within 5 Å from each other was computed to probe the short-range interactions in the fungal cell wall (Fig. 5g). Time-course analysis from triplicate all-atom MD simulations for the sum of all intermolecular contacts between the polysaccharides within 5 Å is included as Supplementary Fig. 20. All system preparation, molecular simulation input, and analysis script are made publicly available on Zenodo.

### Reporting summary

Further information on research design is available in the Nature Portfolio Reporting Summary linked to this article.

### Supplementary information

Supplementary Information

Peer Review File

Description of Additional Supplementary Files

Supplementary Movie 1

Supplementary Movie 2

Supplementary Movie 3

Supplementary Movie 4

Supplementary Movie 5

Reporting Summary

### Source data

Source Data

## Data Availability

All NMR spectra and biochemical data that support the findings of this study are provided in the article and Supplementary Information. The topspin NMR datasets of all ssNMR spectra collected on fungal cell walls are available in the public repository Zenodo under the DOI number: 10.5281/zenodo.12700137. Source data are provided with this paper.

## Code availability

All MD input data, which includes topology, force field parameters and input scripts that support the findings of this research are publicly available via Zenodo under the DOI number 10.5281/zenodo.12700137.
