# Supplementary material for: Adaptative survival of Aspergillus fumigatus to echinocandins arises from cell wall remodeling beyond β−1,3-glucan synthesis inhibition
Source: Nat Commun. 2024 Jul 31;15:6382. doi: 10.1038/s41467-024-50799-8 (PMC11291495; doi:10.1038/s41467-024-50799-8)
Supplement: Supplementary file 1 — Supplementary Information [file 41467_2024_50799_MOESM1_ESM.pdf]

Supplementary Information

Adaptative Survival of Aspergillus fumigatus to Echinocandins Arises from Cell Wall Remodeling Beyond β-1,3-glucan Synthesis Inhibition

Malitha C. Dickwella Widanage[1,#,$], Isha Gautam[1,#], Daipayan Sarkar[2], Frederic Mentink-Vigier[3], Josh V. Vermaas[2,4], Shi-You Ding[5], Andrew S. Lipton[6], Thierry Fontaine[7], Jean-Paul Latgé[8], Ping Wang[9], Tuo Wang[1*]

[1] Department of Chemistry, Michigan State University, East Lansing, MI, USA

[2] MSU-DOE Plant Research Laboratory, East Lansing, MI, USA

[3] National High Magnetic Field Laboratory, Tallahassee, FL, USA

[4] Biochemistry and Molecular Biology, Michigan State University, East Lansing, MI, USA

[5] Department of Plant Biology, Michigan State University, East Lansing, MI, USA

[6] Environmental Molecular Sciences Laboratory, Pacific Northwest National Laboratory, Richland, WA, USA

[7] Institut Pasteur, Université Paris Cité, INRAE, USC2019, Unité Biologie et Pathogénicité Fongiques, F-75015 Paris, France

[8] Institute of Molecular Biology and Biotechnology, University of Crete, Heraklion, Greece

[9] Departments of Microbiology, Immunology and Parasitology, Louisiana State University Health Sciences Center, New Orleans, LA, USA

[$] Current address: National High Magnetic Field Laboratory, Tallahassee, FL, USA

[#] These authors contributed equally

* Correspondence and requests for materials should be addressed to T.W.

(Email: wangtuo1@msu.edu)

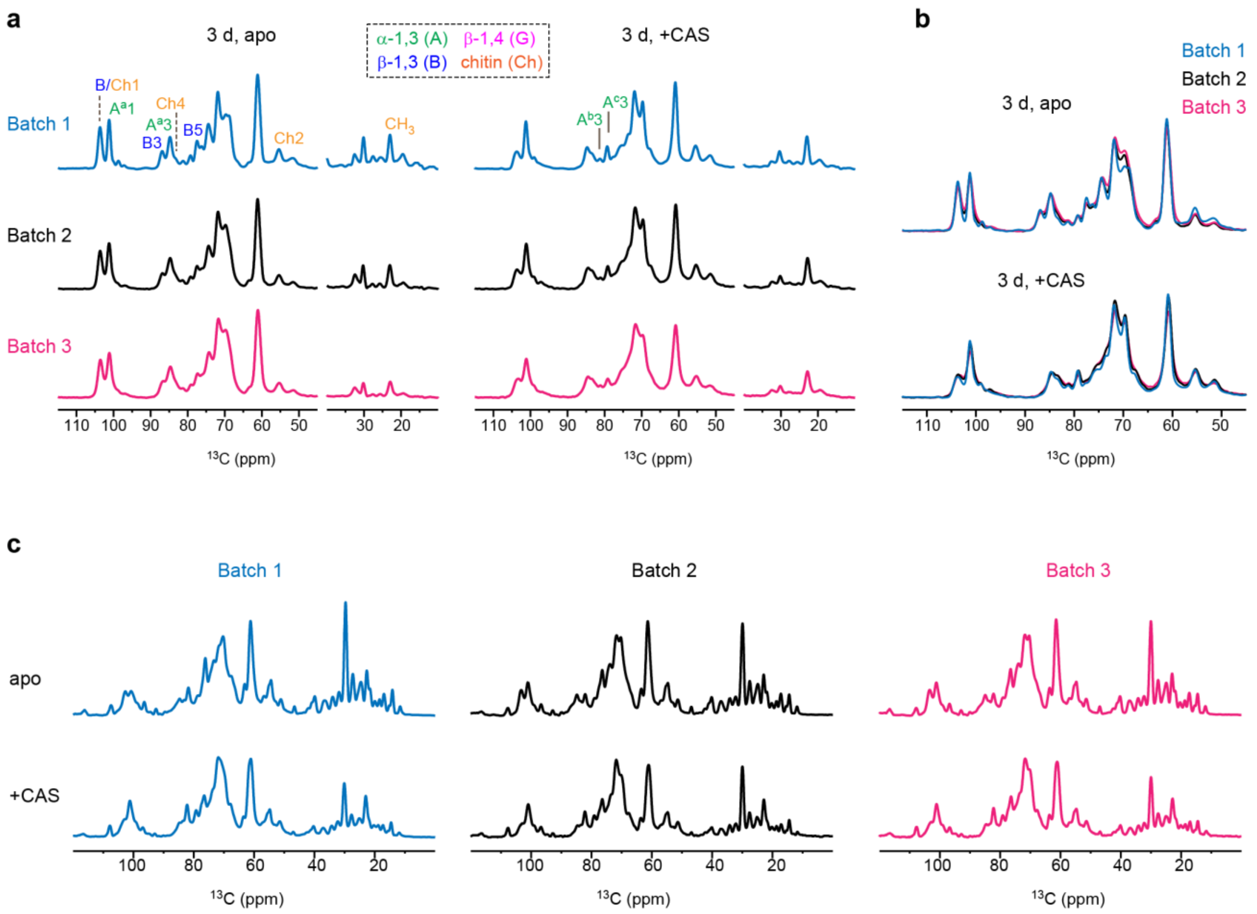

Supplementary Figure 1. Replication of *A. fumigatus* samples under caspofungin treatment. a, 1D ^{13}C CP spectra of 3-day-old sample without drug (left) and with drug (right). **b**, Overlay of the CP spectra of three sample batches. The overall spectral patterns are comparable, exhibiting slight fluctuations in peak intensities and linewidths. **c**, Quantitative ^{13}C DP spectra (with a recycle delay of 35 s) of 3-day-old without drug (top) and with drug (bottom). The carbohydrate region is highly replicable. The only noticeable change occurs at the 32-ppm peak, which is the lipid acyl chain (CH_2).

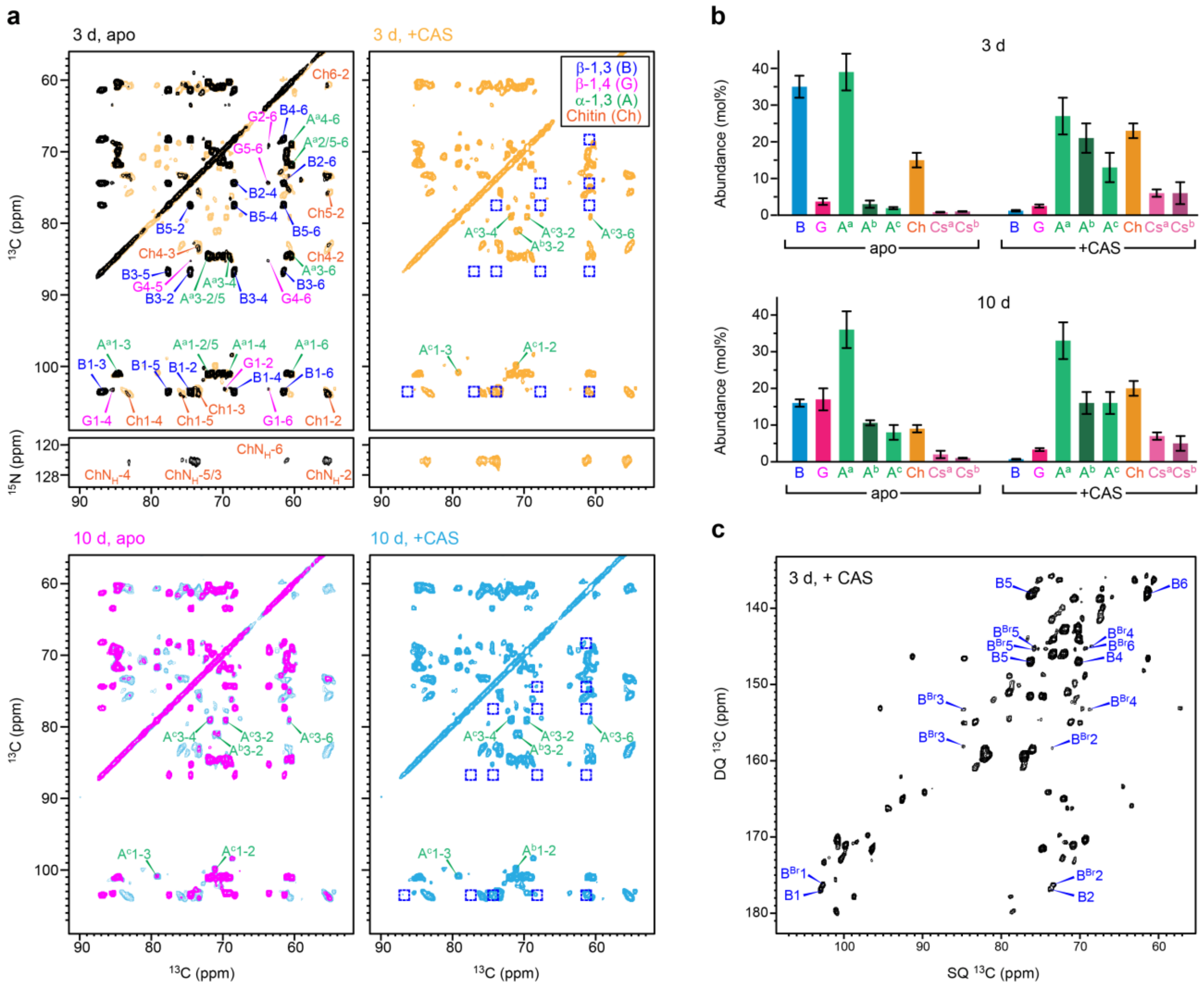

Supplementary Figure 2. Effect of caspofungin and culture duration on cell wall composition. a, 2D ^{13}C-^{13}C/^{15}N spectra of 3-day-old (top) and 10-day-old (bottom) *A. fumigatus* cell walls without and with caspofungin (CAS). The spectra were measured using CP and 53-ms CORD mixing for detecting chitin and glucans in the rigid portion of *A. fumigatus* cell walls. The ^{15}N-^{13}C correlation spectra were measured using NCACX sequence, showing correlations between chitin N_H and carbons. In the first column, the spectra of drug-treated and apo samples were overlaid for comparison. Abbreviations are used for resonance assignment and different polysaccharide signals are color-coded. The missing peaks of β-1,3-glucan linkage in drug-treated cell walls is marked using blue squares. **b**, Molar composition of cell wall polysaccharides. The compositional data was obtained by analysis of the intensities of resolved cross peaks in 2D spectra. Error bars represent standard error derived from the intensities of the different cross-peaks for each type of polysaccharides. **c**, 2D ^{13}C DP J-INADEUQATE spectrum of 3-day-old drug-treated *A. fumigatus* resolves the signals from the linear chain (B) and the branching points (B^{Br}) of β-glucans. Source data are provided as a Source Data file.

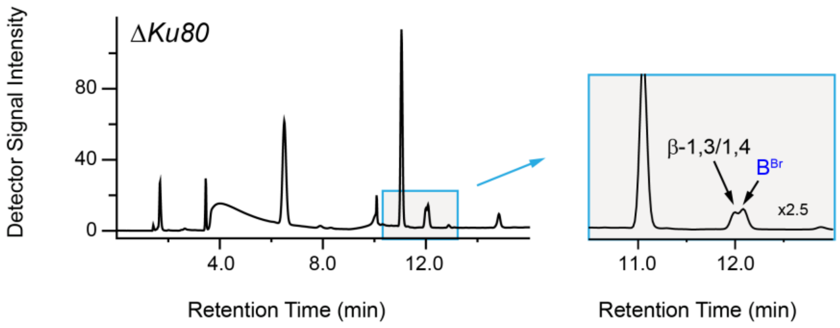

Supplementary Figure 3. Structure of β-glucans from chemical analysis. Data obtained from the Δ*akuB*^{KU80} strain, which of is another widely used model strain of *A. fumigatus*, allow us to validate the results obtained on the wild-type strain used in this study. The right column shows the zoom-in regions where the different types of β-linkages could be resolved.

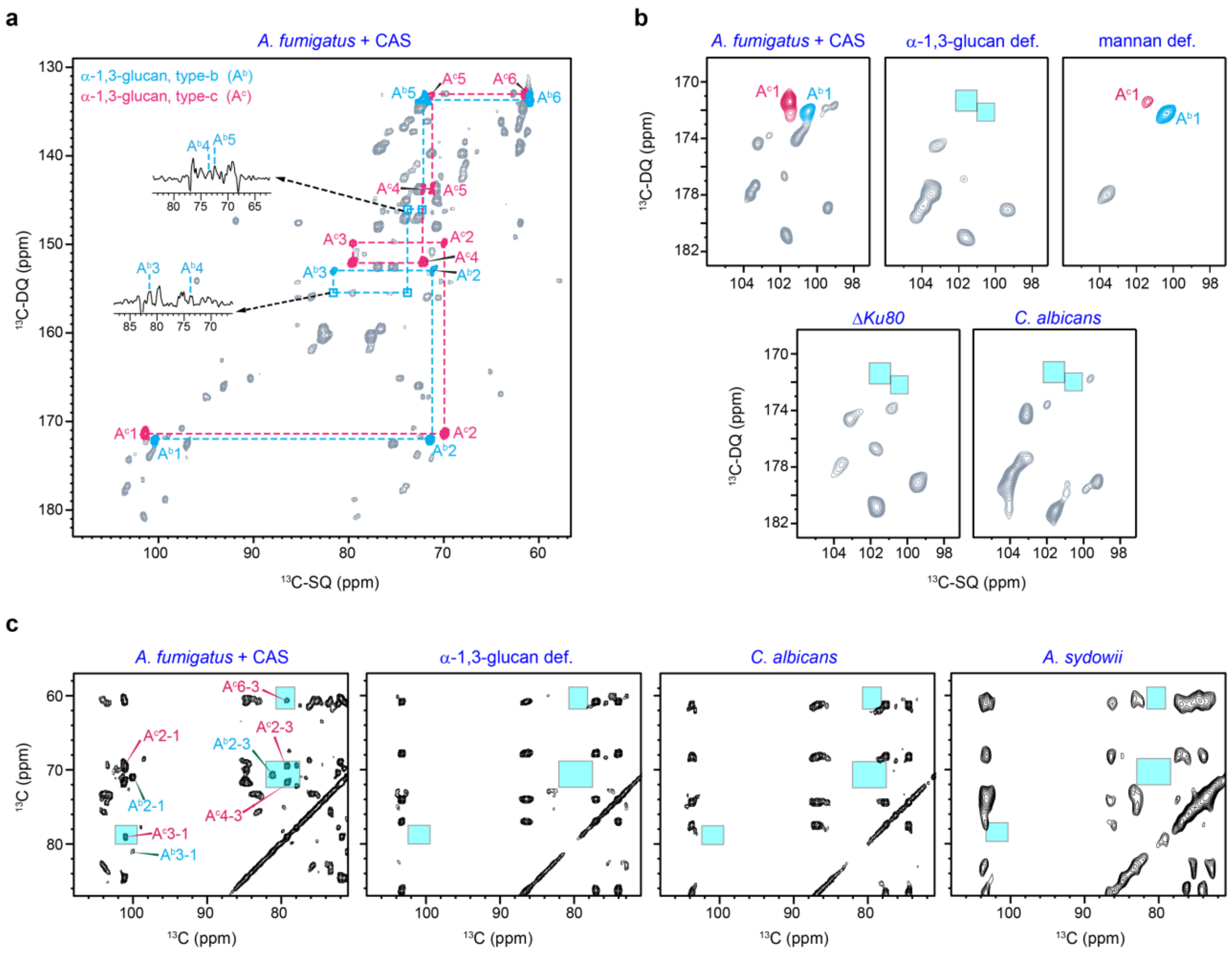

Supplementary Figure 4. Identification of two minor forms of α-1,3-glucans. a, Carbon connectivity of type-b (A^b; cyan) and type-c (A^c; magenta) forms of α-1,3-glucans in the caspofungin-treated *A. fumigatus* identified in 2D ^{13}C DP J-INADEUQATE spectra. Cross-sections are shown for signals that are weak and below the plotted contour level. **b**, Comparison of the carbon-1 regions of DP J-INADEUQATE spectra confirms the identity of the two minor forms of α-1,3-glucans and their presence in the mobile portion of cell wall polysaccharides. The type-b and type-c forms of α-1,3-glucans are resolved in the drug-treated cell walls of wild-type *A. fumigatus* (RL578) but absent in the α-1,3-glucan-deficient mutant, thus confirming the identity and assignment of these signals. Cyan boxes are used to highlight the missing signals of the minor forms of α-1,3-glucans. The signals are also absent in the mannan-deficient mutant, thus excluding the attribution from the manna polymers that dominate this type of spectra. The signals are absent in a widely used model strain Δ*akuB*^{KU80} of *A. fumigatus*, and in *Candida albicans*, evidencing their unique abundance in the caspofungin-treated A. fumigatus. **c**, Confirmation of the presence of type-b and type-c forms of α-1,3-glucans in the rigid portion of caspofungin-treated A. fumigatus cell walls as detected using 53-ms CORD spectrum. The corresponding signals are confirmed to be absent in α-1,3-glucan-deficient mutant of A. fumigatus, as well as in *C. albicans*, and *A. sydowii*. All 2D ^{13}C-^{13}C correlation spectra were collected on an 800 MHz ssNMR spectrometer, except the spectrum of *A. sydowii*, which was measured on a 400 MHz NMR instrument.

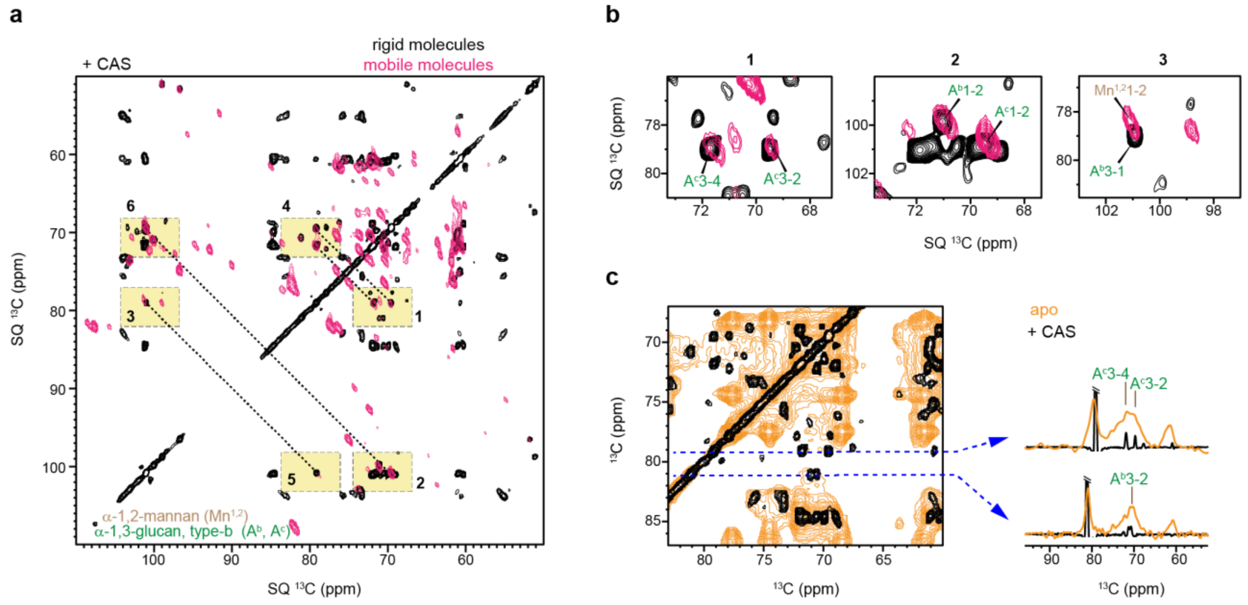

Supplementary Figure 5. Distribution of minor forms of α-1,3-glucans in dynamically distinct domains. a, Overlay of two 2D ^{13}C-^{13}C correlation spectra of 3-day-old *A. fumigatus* treated with caspofungin: the CP-based 53-ms CORD spectrum (black) selecting rigid molecules and the sheared DP-based refocused J-INADEQUATE spectrum (magenta) selecting mobile components. Both spectra exhibit signals of the type-b (A^b) and type-c (A^c) of α-1,3-glucans in six highlighted regions. **b**, Zoomed-in regions extracted from panel a, confirming that the type-b and type-c α-1,3-glucan are distributed in both rigid and mobile fractions of drug-treated *A. fumigatus* cell walls. **c**, Type-c of α-1,3-glucan is also present in the drug-free *A. fumigatus* cell walls but with low abundance, which is shown by the overlay of 53-ms CORD spectra of 3-day-old cell wall with (orange) and without (black) caspofungin. The cross-sections extracted at 81 ppm and 79 ppm show the signals of type-b and type-c forms, respectively. The spectrum of apo sample is processed with more line-broadening to show the weaker signals of these two minor forms of α-1,3-glucans in untreated *A. fumigatus.* All spectra were collected on an 800 MHz NMR under 12 kHz MAS.

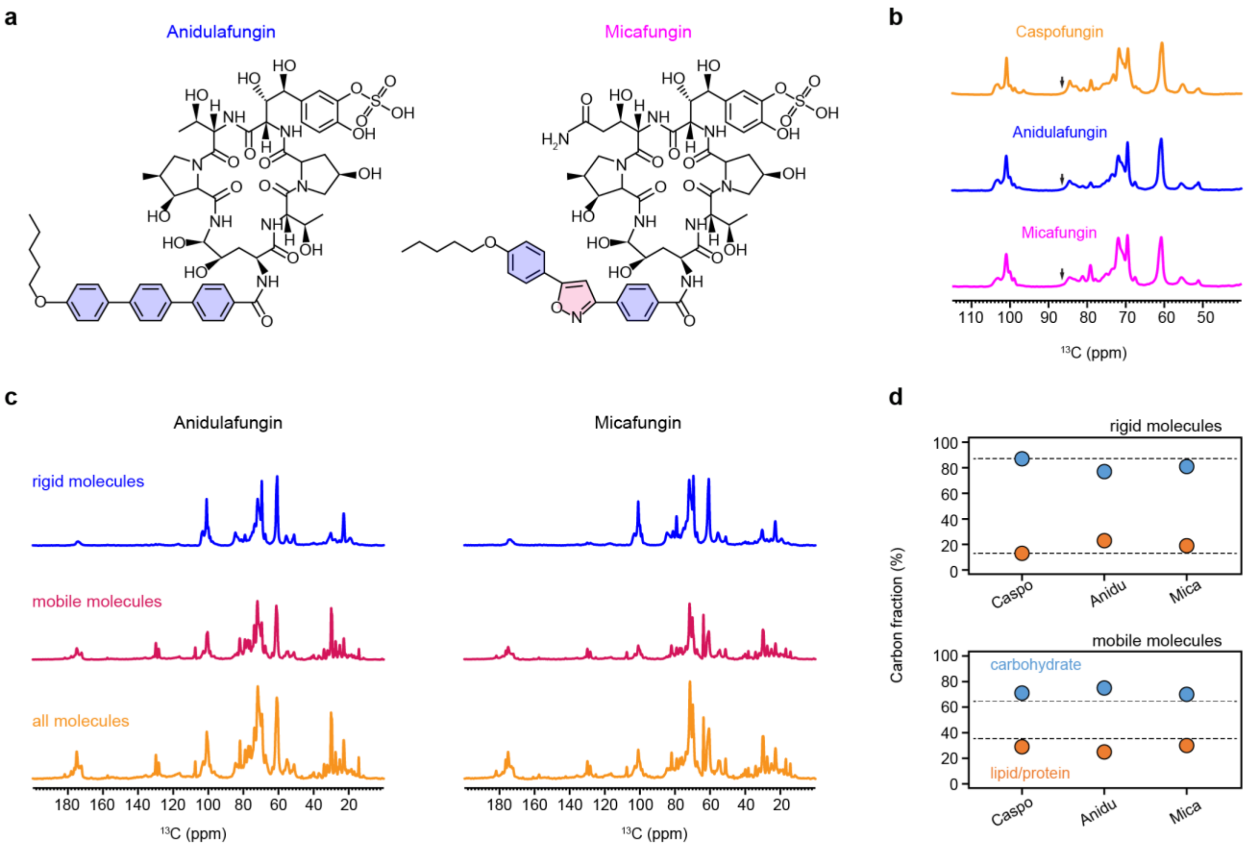

Supplementary Figure 6. Effects of different echinocandins on *A. fumigatus*. a, Chemical structures of anidulafungin and micafungin, with difference in the lipid tail structure. **b**, 1D ^{13}C CP spectra of 3-day-old *A. fumigatus* samples treated with caspofungin (top), anidulafungin (middle), and micafungin (bottom), showing highly similar carbohydrate signals. The well-resolved signals of β-glucans, such as B3 at 86-87 ppm (arrows), are absent in all three samples. **c**, 1D ^{13}C NMR spectra of anidulafungin-treated (left) and micafungin-treated (right) *A. fumigatus* samples. These spectra were measured using CP for detection of rigid molecules (top), DP and 2 s recycle delays for detecting mobile molecules (middle), as well as DP and 35 s recycle delays for quantitative detection of all molecules (bottom). **d**, Quantification of the total carbon fraction (%) of carbohydrate (blue) and lipid/protein (orange) within each sample. Dashed line indicates the corresponding values of the apo 3-day-old sample. None of the echinocandins cause major perturbation to the total carbohydrate content nor the total lipid/protein content. Source data are provided as a Source Data file.

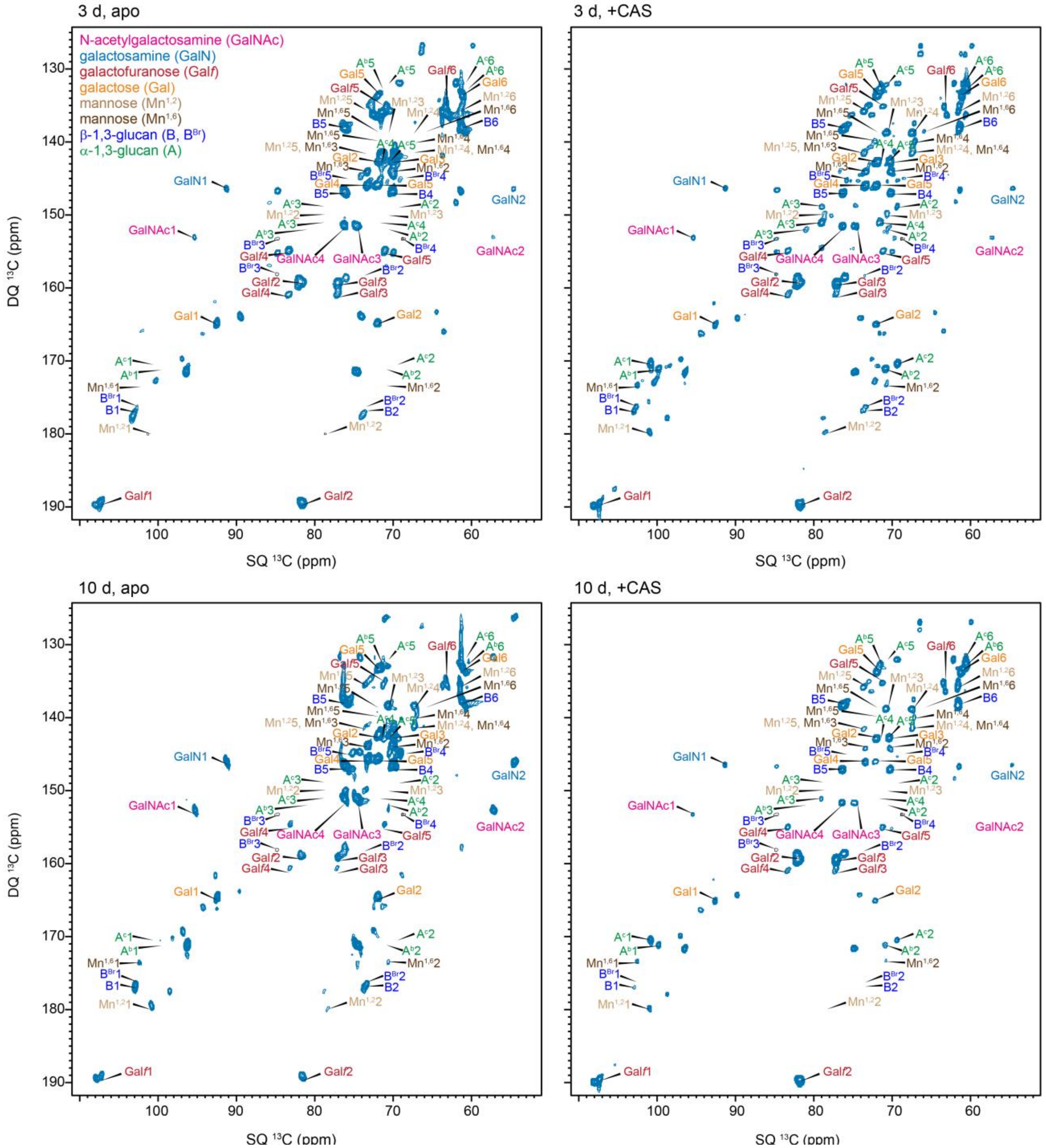

Supplementary Figure 7. Mobile domains containing heteropolysaccharides of GAG and GM. GAG contains N-acetylgalactosamine (GalNAc), galactosamine (GalN), and galactose (Gal) residues. GM contains galactofuranose (Gal*f*) and mannose residues with 1,2- and 1,6-linkages ($Mn^{1,2}$ and $Mn^{1,6}$). In both polysaccharides, sugar residues are arranged in no particular order. B^{Br} indicates branching point in the β-(1,3)-linked backbone. Their chemical shifts are consistent in all four samples. The ^{13}C DP refocused J-INADEQUATE spectra detect the ^{13}C through-bond connectivity for mobile polysaccharides. All spectra were measured on 800 MHz NMR spectrometer at 12 kHz MAS.

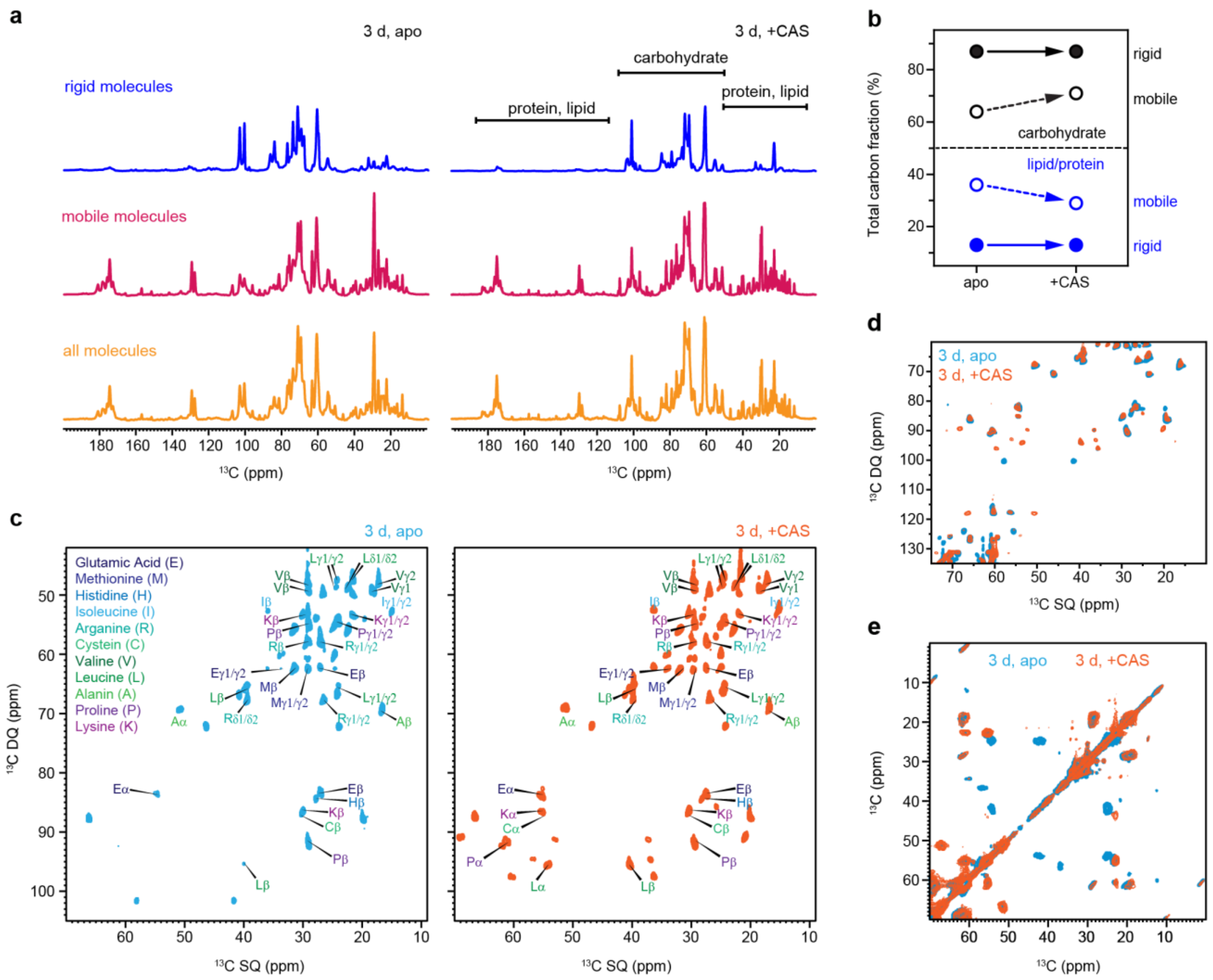

Supplementary Figure 8. Protein components are retained after caspofungin treatment. **a**, 1D ^{13}C NMR spectra measured using different pulse sequences: 1D ^{13}C CP for detecting rigid molecules, 2 s DP for detecting mobile molecules, and 30 s DP for quantitatively detecting all molecules. The spectra were collected from a 3-day-old sample both without (left) and with (right) caspofungin treatment. Specific regions corresponding to polysaccharides and protein/lipids were highlighted. **b**, Quantification of the total carbon fraction (%) of polysaccharides (black) and lipid/protein (blue) content within the sample. The sample fractions within mobile and rigid phases are indicated by open and filled circles, respectively. The alteration in the protein/lipid to polysaccharide ratio was observed exclusively in the mobile phase, with a minor change of less than 10%. **c**, 2D ^{13}C DP refocused J-INADEQUATE spectra showing signals of mobile proteins, with highly overlapping signals that indicate similar protein structures. **d**, Overlay of protein Cα-Cβ signals in 2D DP J-INADEQUATE spectra of apo (cyan) and caspofungin-treated (orange) samples. Both spectra are processed with increased line broadening (LB of -30 and GB of 0.03) to enhance the sensitivity of protein signals. **e**, Overlay of two 2D ^{13}C-^{13}C 53 ms CORD correlation spectra of the rigid proteins in apo (cyan) and caspofungin-treated (orange) samples. All spectra were measured on 800 MHz NMR spectrometer at 12 kHz MAS. Source data are provided as a Source Data file.

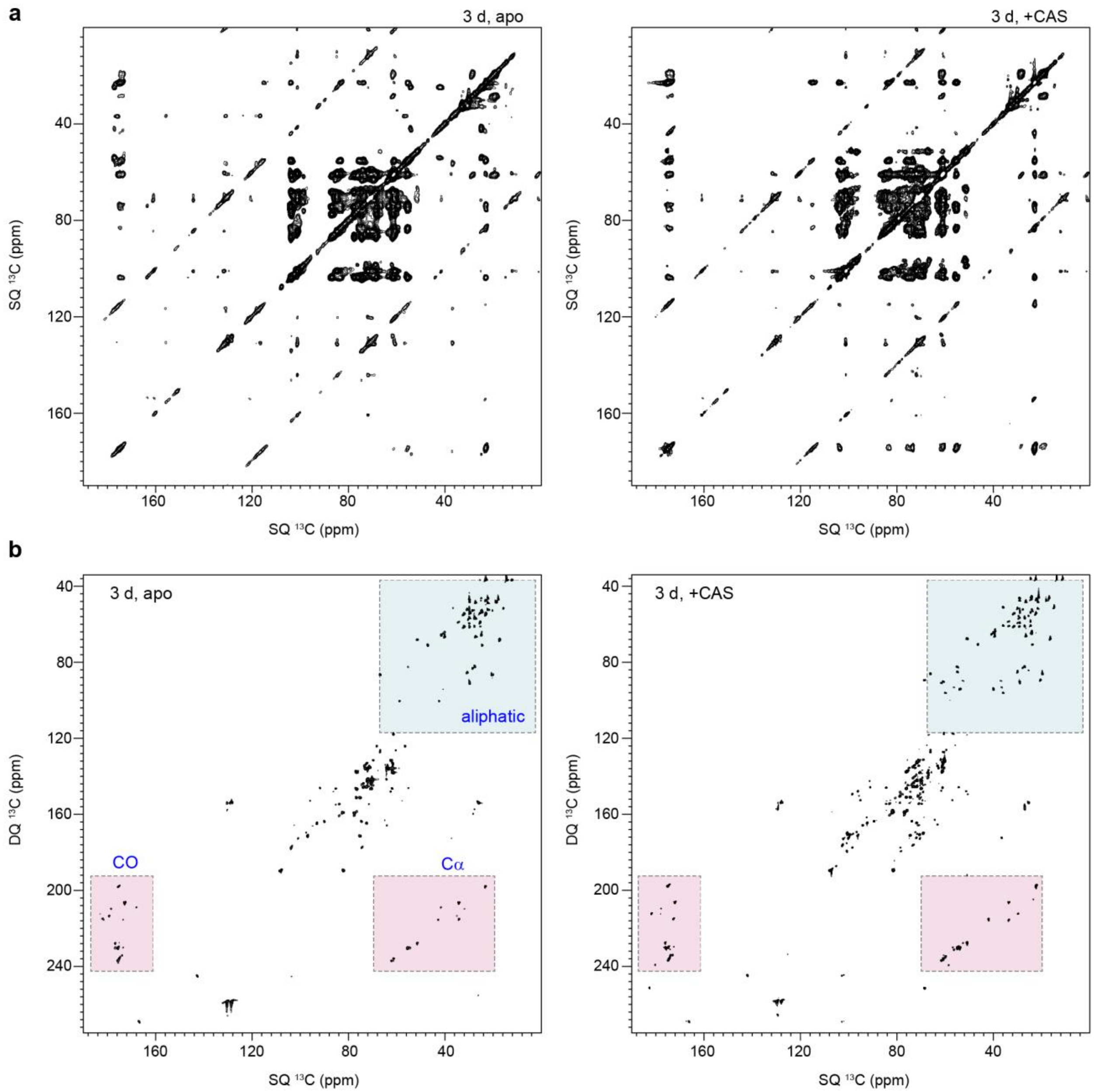

Supplementary Figure 9. Full-range 2D ^{13}C-^{13}C correlation spectra of *A. fumigatus* samples. **a**, 2D ^{13}C-^{13}C 53 ms CORD spectra of 3-day-old apo (left) and caspofungin-treated (right) samples. **b**, Full 2D DP J-INADEQUATE spectra of 3-day-old apo (left) and caspofungin-treated (right) samples. The representative spectral regions of aliphatic, CO, and Cα are highlighted.

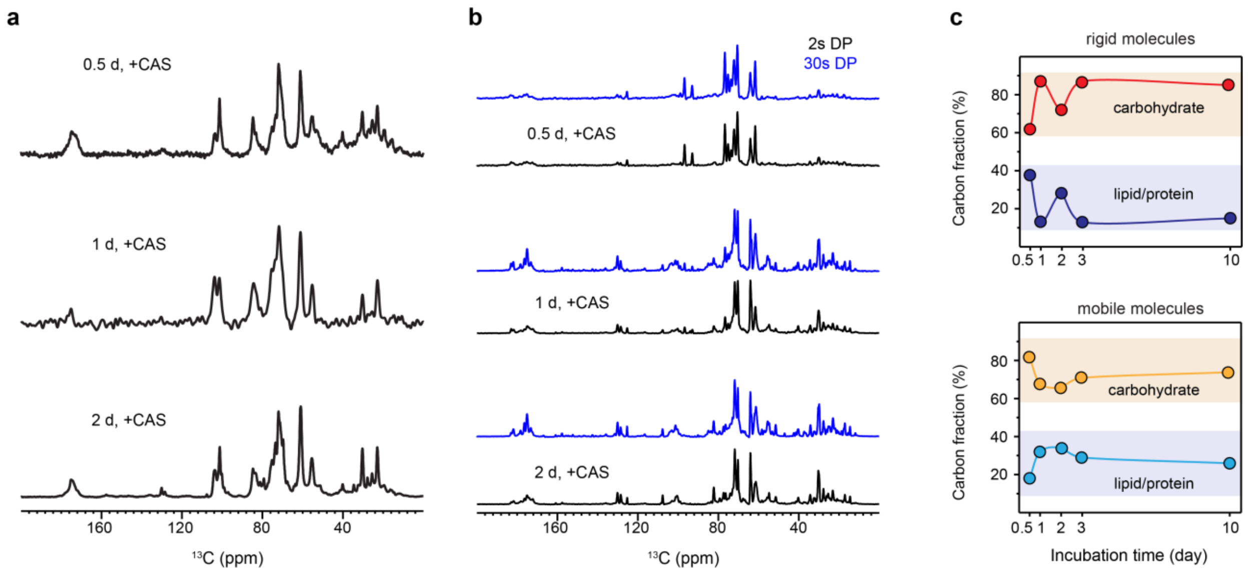

Supplementary Figure 10. Proteins, lipids, and carbohydrates in younger cultures. **a**, 1D ^{13}C CP spectra of caspofungin-treated A. fumigatus samples cultured for 0.5 day (top), 1 day (middle), and 2 days (bottom), with the spectra selectively identifying rigid molecules. **b**, 1D DP spectra measured using different recycle delays: 2 s for detecting mobile molecules (black) and 30 s (blue) for quantitative detection of all molecules. **c**, Quantification of the total carbon fraction (%) of carbohydrates and lipid/protein over culture time. The top and bottom panels depict the carbon fractions in the rigid and mobile phases, respectively.

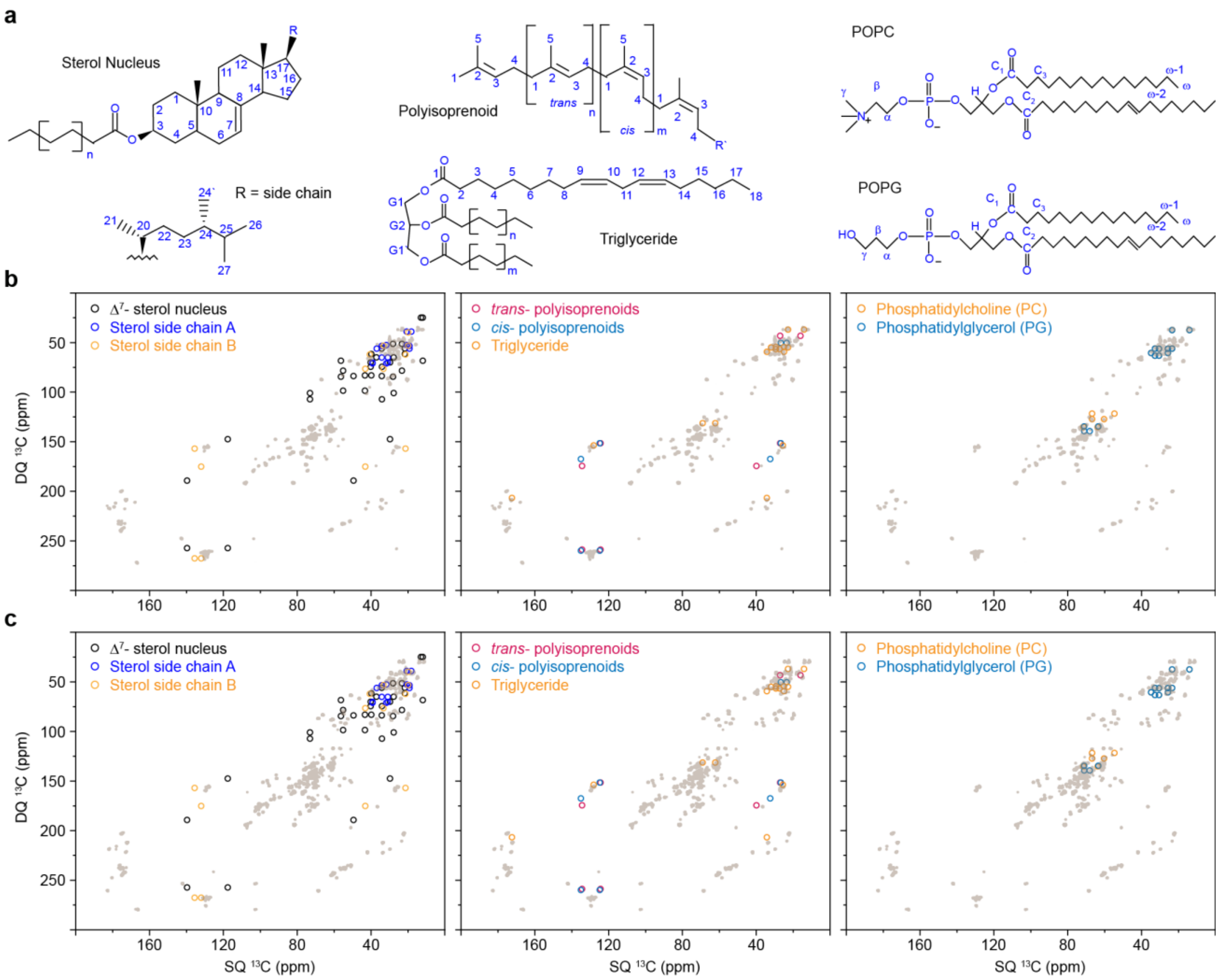

Supplementary Figure 11. Possible membrane and lipid components. a, Possible structures of lipid components (sterol, polyisoprenoid, and triglycerides) and chemical structures of model phospholipids POPC and POPG. **b**, Overlay of 2D DP refocused J-INADEQUATE spectra of 3-day-old apo sample (gray) with simulated spectra. **c**, Overlay of 2D DP refocused J-INADEQUATE spectra of 3-day-old caspofungin-treated sample (gray) with simulated spectra. The simulated spectra were plotted using the chemical shifts reported in recent NMR studies[1-5].

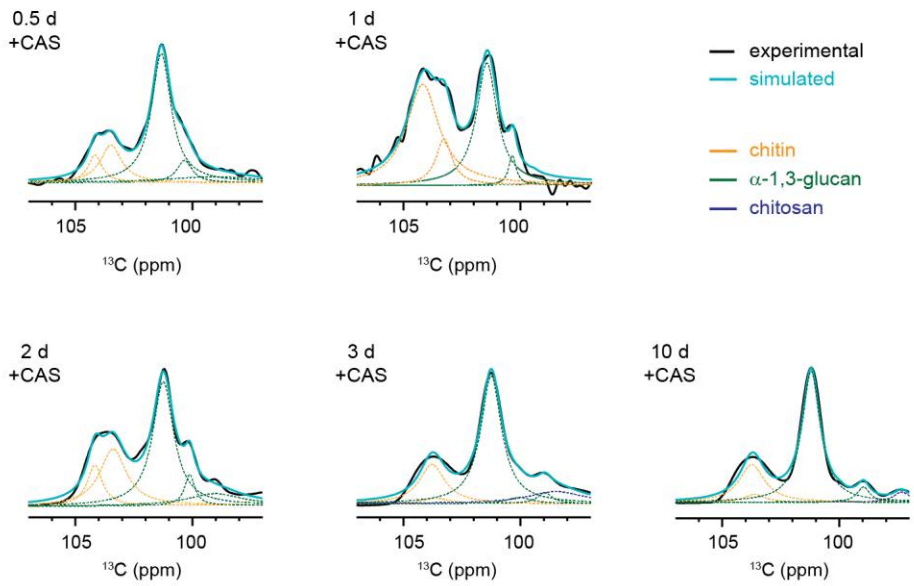

Supplementary Figure 12. Spectral deconvolution of C1 region. Carbon-1 regions of 1D CP spectra show changes in carbohydrate composition across various culture durations. The spectra were deconvoluted to unveil the resonances of distinct carbohydrate components, and the simulated data exhibit a close match to the experimentally measured spectra.

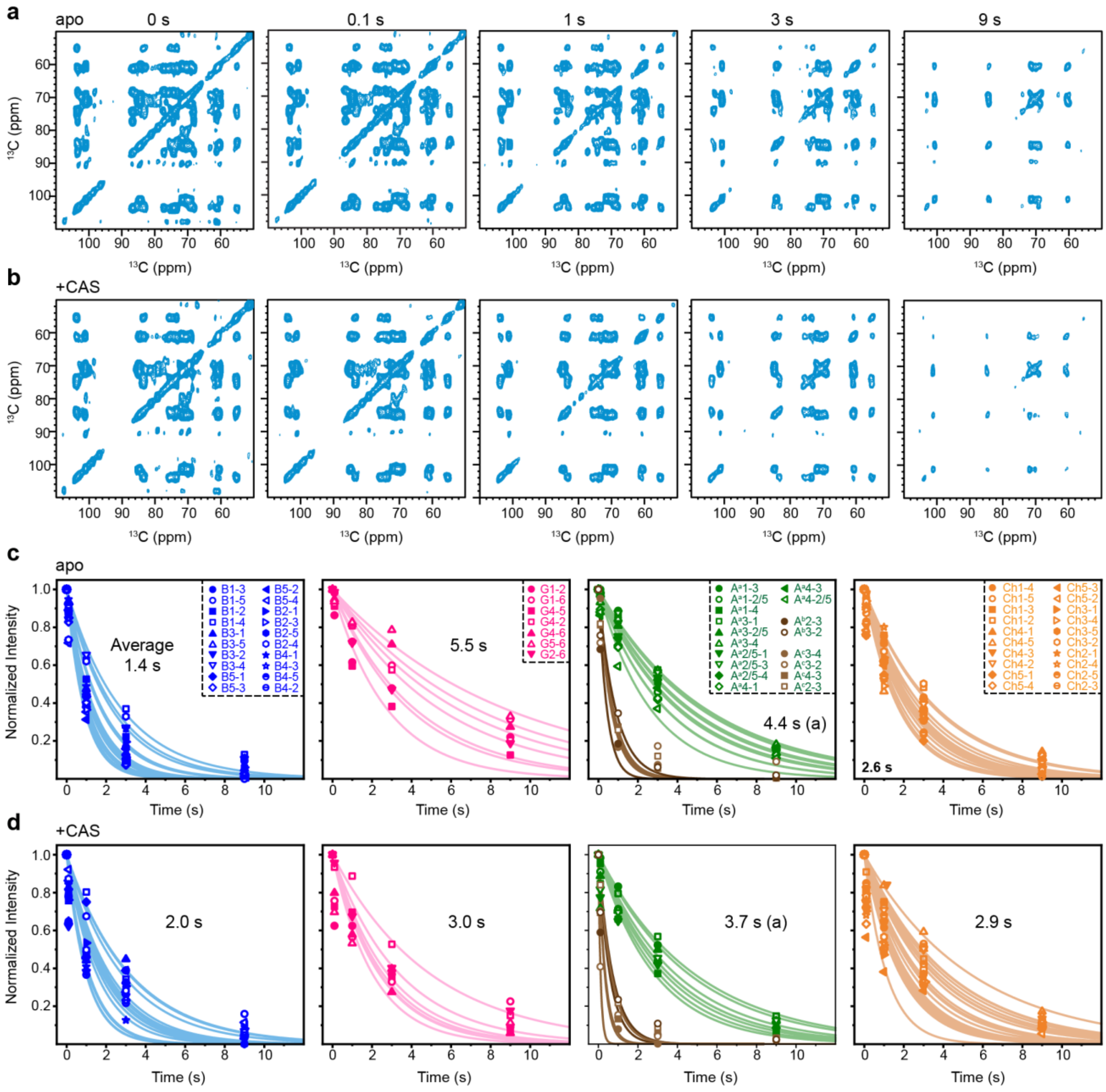

Supplementary Figure 13. ^{13}C-T$_1$ relaxation data of 3-day-old *A. fumigatus* cell walls. Two arrays of 2D ^{13}C-^{13}C spectra are shown for **a**, the *apo* sample and **b**, caspofungin-treated cell walls. From left to right, the z-filter duration increases (0 s, 0.1 s, 1 s, 3 s, and 9 s), and the intensities decrease. The intensity decay was plotted as a function of z-filter time for each carbon site. The relaxation decay curves are shown separately for **c,** the apo sample and **d,** the caspofungin-treated sample. The average ^{13}C-T$_1$ time constants of polysaccharides are labeled. For α-1,3-glucan, the type-a subform is shown as green while the type-b and c forms in brown, with significantly faster relaxation. Source data are provided as a Source Data file.

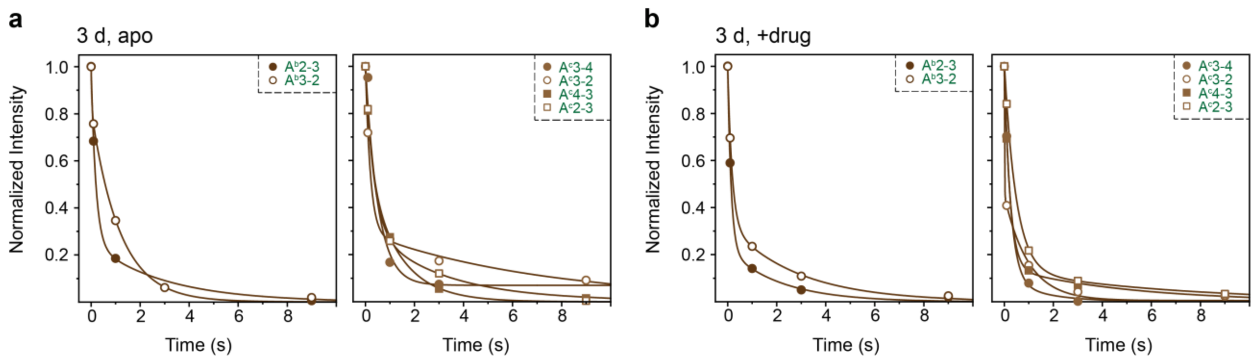

Supplementary Figure 14. Double-exponential ^{13}C-T$_1$ relaxation of new α-glucan forms. Most signals of type-b and type-c α-1,3-glucan in both the **a**, apo and **b**, caspofungin-treated 3-day-old A. fumigatus samples fit better with double-exponential equations, revealing their distribution in dynamically different domains within the cell wall. Source data are provided as a Source Data file.

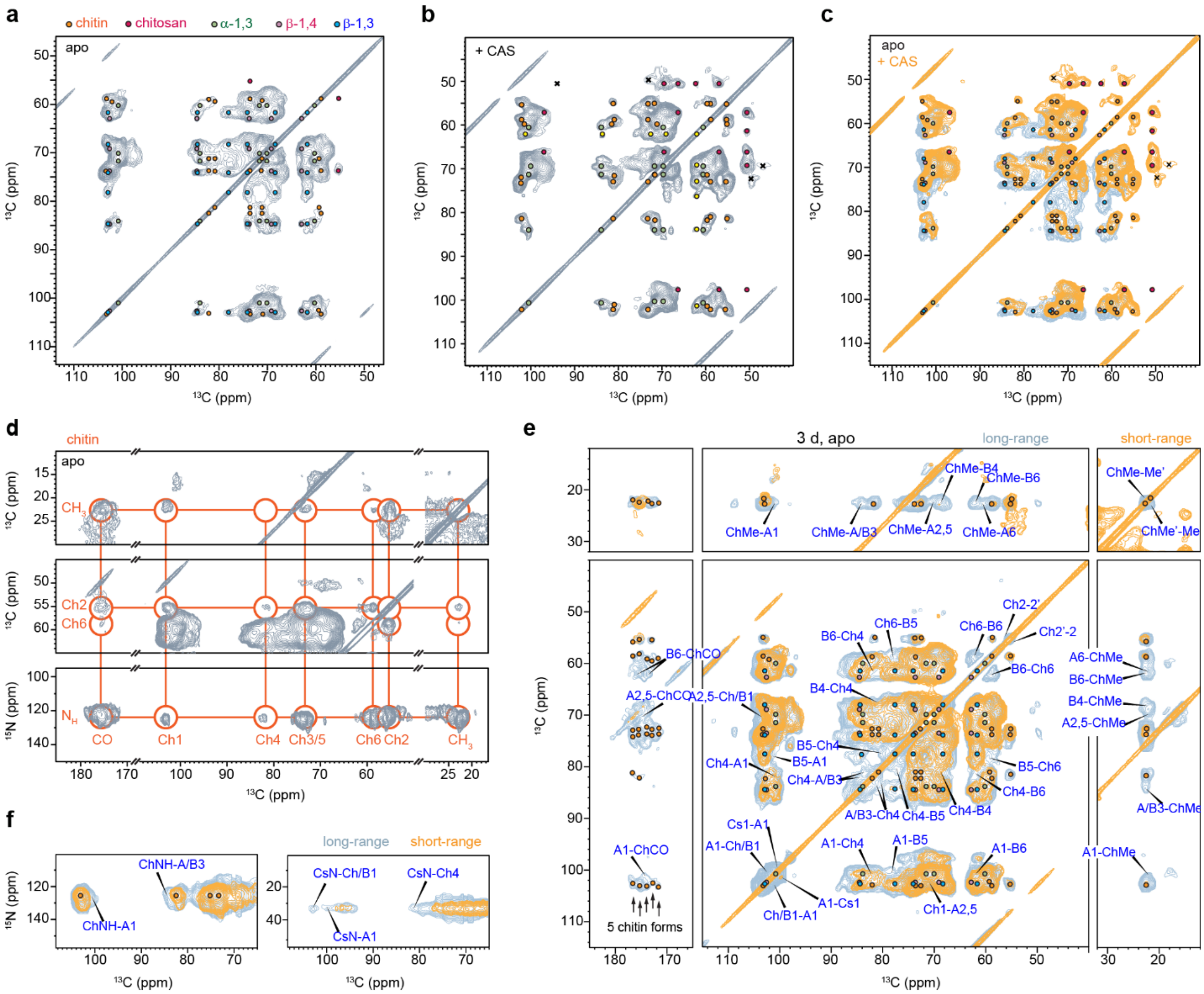

Supplementary Figure 15. DNP analysis of cell wall polysaccharides and packing interface. Carbohydrate resonance assignment was shown for **a**, apo and **b**, drug-treated 3-day-old samples. The DNP 2D ^{13}C-^{13}C spectra were measured using 100-ms PDSD mixing. **c**, Overlay of 2D ^{13}C-^{13}C spectra of 3-day-old apo (grey) and drug-treated (yellow) samples showing the removal of β-1,3-glucan after CAS treatment. **d**, DNP 2D ^{15}N/^{13}C-^{13}C correlation spectra show the carbon and nitrogen connectivity in chitin in the apo sample. **e**, Intermolecular interactions of 3-day-old cell wall detected by overlaying 100-ms PDSD (short range; yellow) and 20-ms PAR (long range; grey) spectra under DNP enhancement. **f,** ^{15}N-^{13}C correlation spectra of 3-day-old apo sample measured with 100-ms (yellow) and 3-s (grey) ^{13}C-^{13}C mixing periods. The cross peaks are between chitin -NH- (left panel) and chitosan -NH_2 (right panel) with carbohydrate carbon sites. All spectra were collected on a 600 MHz/395 GHz MAS-DNP spectrometer.

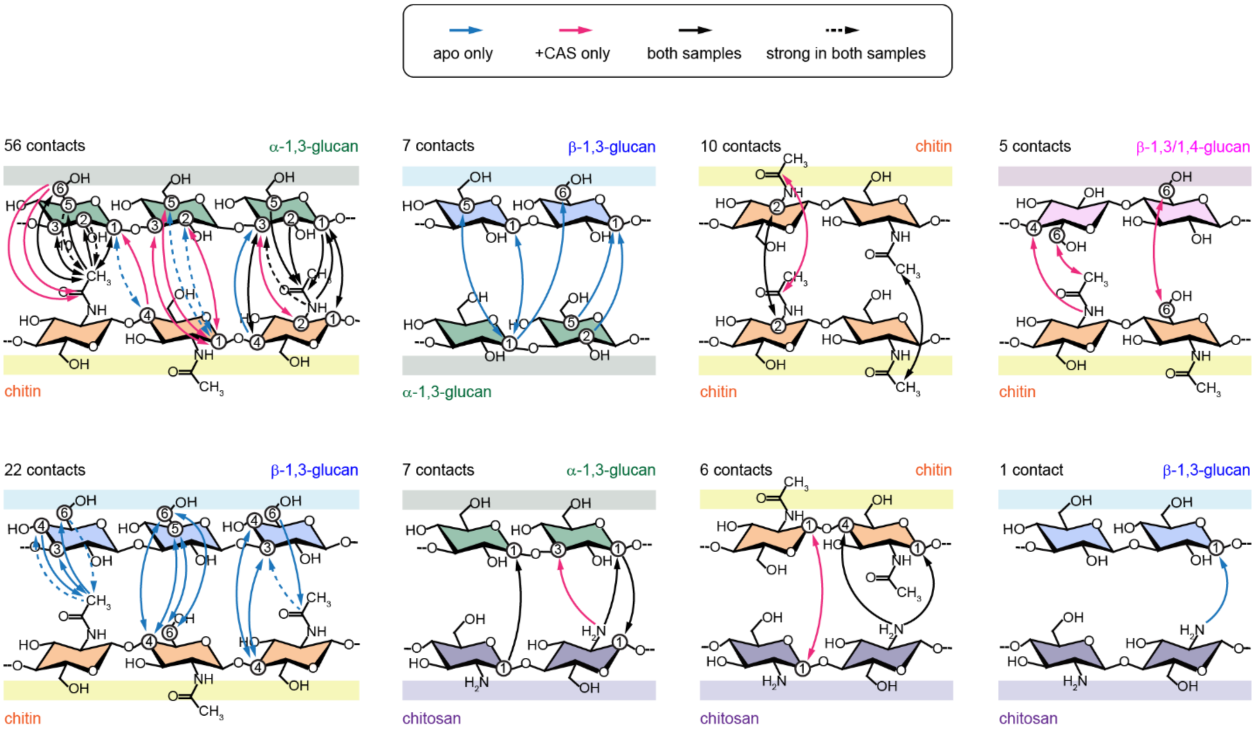

Supplementary Figure 16. Structural summary of NMR-observed intermolecular contacts. The 114 intermolecular cross peaks identified in 3-day-old *A. fumigatus* cell walls are categorized based on polysaccharide species at the packing interface. Interactions unique to the apo sample are depicted by blue solid lines, those exclusive to drug-treated samples by magenta solid lines, and shared interactions by black solid lines. Strong cross peaks observed in short-mixing 2D correlation spectra and present in both apo and drug-treated samples are indicated by black dashed lines. Arrowheads denote the direction of polarization transfer between carbon and nitrogen sites, encompassing both one-way (one cross peak) and bidirectional transfers (two cross peaks). The number of cross peaks observed between each pair of polysaccharides is indicated in each figure panel.

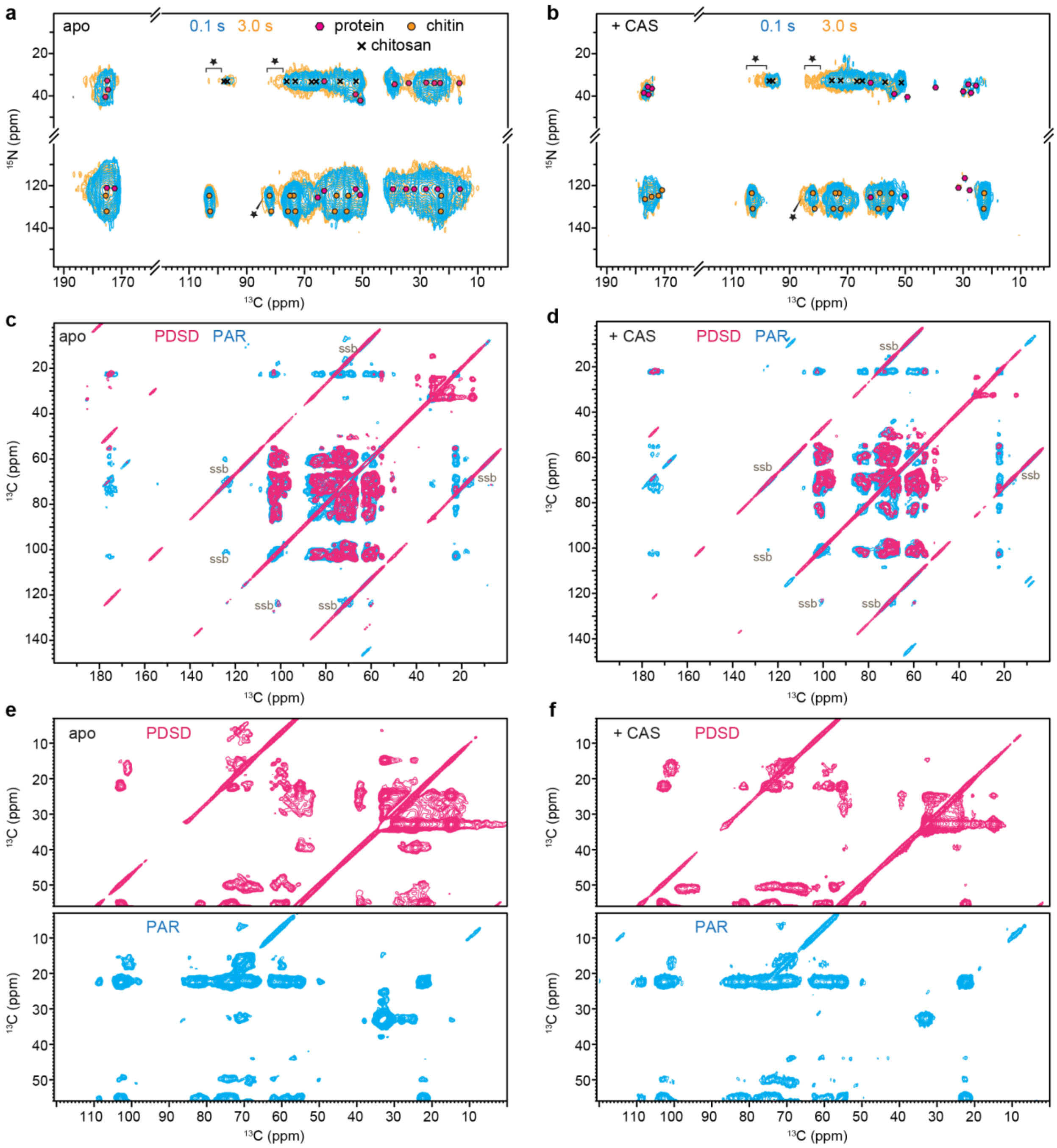

Supplementary Figure 17. DNP detection of proteins and lipids in 3-day-old *A. fumigatus*. 2D ^{15}N-^{13}C correlation spectra of **a**, apo and **b**, drug-treated 3-day-old *A. fumigatus* samples exhibit signals from chitin NH (~125-135 ppm), chitosan NH_2 (~33 ppm), and some protein amide (~35-45 ppm). Overlay of spectra with 0.1-s and 3.0-s mixing times detect intra and inter-molecular cross peaks Asterisks denote intermolecular cross peaks. 2D ^{13}C-^{13}C correlation spectra of **c**, apo and **d**, drug-treated samples did not show clear cross peaks between protein/lipid and polysaccharides. Zoomed-in regions are shown for **e**, apo and **f**, caspofungin-treated samples, showing signals of proteins/lipids and carbohydrates, yet lacking intermolecular cross peaks between them.

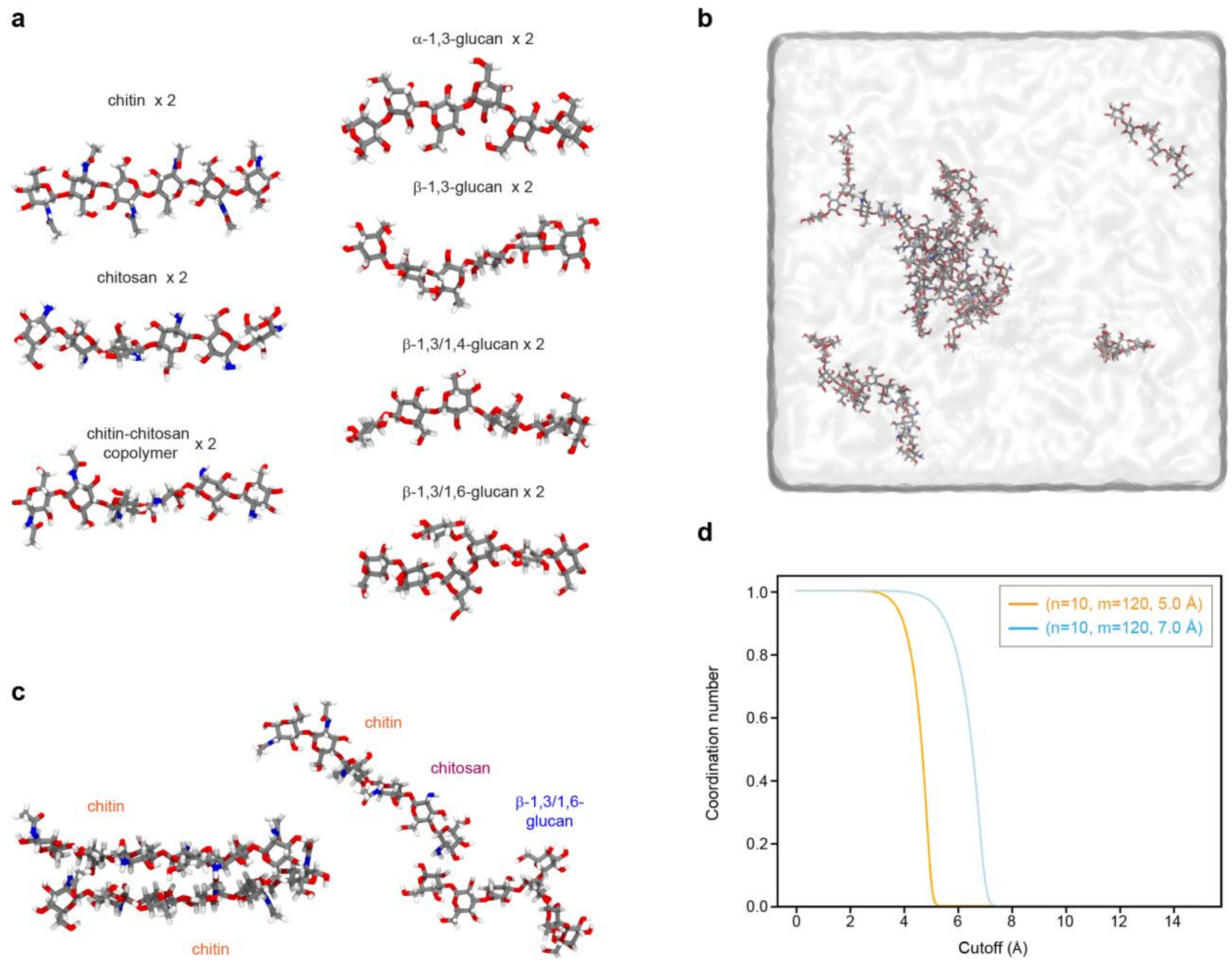

Supplementary Figure 18. Atomic models used for all-atom MD modeling. a, Atomic model of individual components included in the fungal cell wall molecular system. In total 14 molecules were included in the modeling, with two copies for each of the seven types of polysaccharides: chitin, chitosan, chitin-chitosan copolymer, α-1,3-glucan, linear β-1,3-glucan, branched β-1,3/1,6-glucan, and terminal β-1,3/1,4-glucan. **b,** Assembly of the molecular system solvated with water. Molecular structures for different polymers using VMD 1.9.4. The atomistic model consists of 120, 972 atoms after adding solvent and ions. **c,** Short-range interactions between two chitin chains, and between chitin-chitosan copolymer and β-1,3-glucan observed during 1 μs all-atom MD. **d,** Coordination number for short-range and long-range cutoff distance of 5.0 and 7.0 Å respectively, with the exponents n =10 and m = 120. See Methods for mathematical expression.

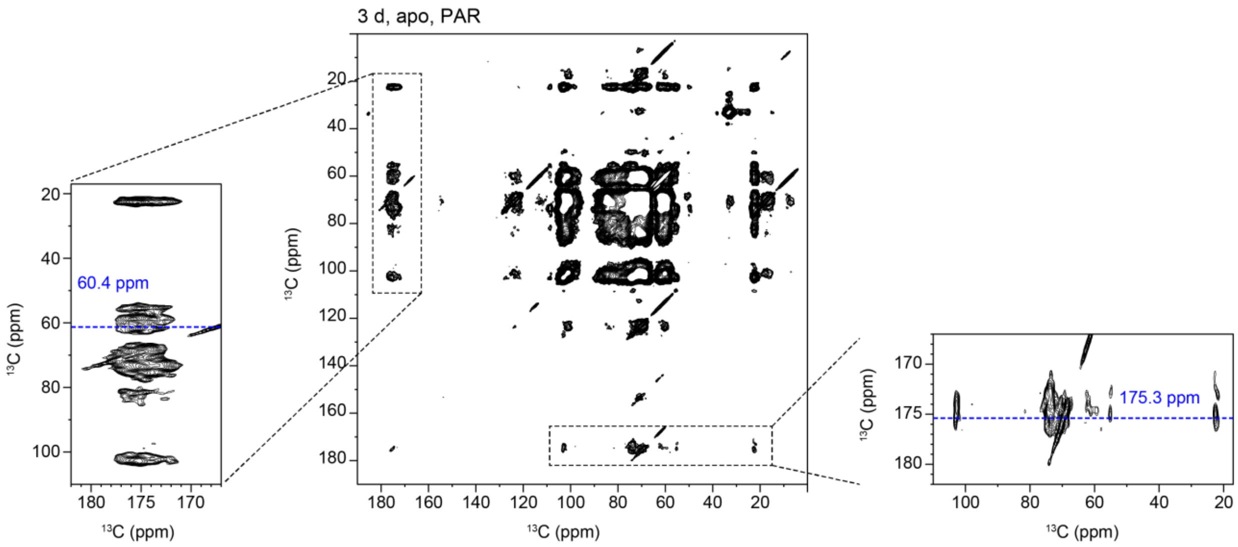

Supplementary Figure 19. Spectral asymmetry observed for intermolecular cross peaks. Zoom-in views are provided for spectral regions involving interactions between chitin carbonyl and other polysaccharides. The A6-ChCO cross peak appears strong due to A6 (ω_1=60.4 ppm) being a protonated carbon site. However, the expected reverse cross peak, ChCO-A6, did not show strong signals, because of polarization initiation from the non-protonated CO site at ω_1=175.3 ppm.

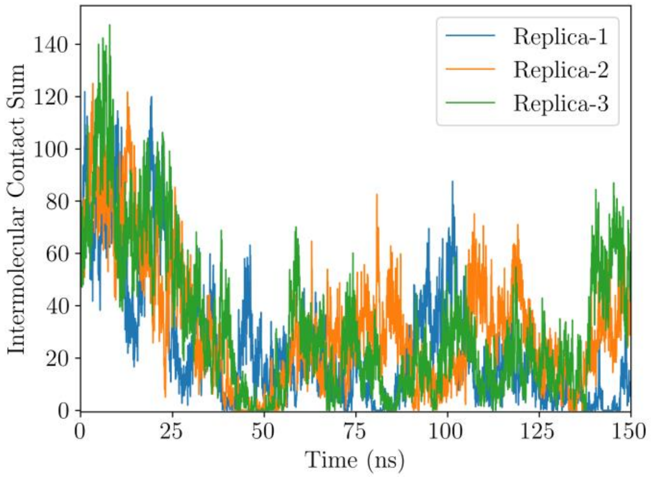

Supplementary Figure 20. Time-course analysis and convergence in all-atom MD simulation. Sum of total intermolecular contacts between the polysaccharides in the fungal cell wall, considering a 5.0 Å cutoff. Triplicate simulations as indicated indicate similar contact profiles. The figure here represents the sum of total intermolecular contacts between cell wall polysaccharides, $\sum_{i,j} C_{i,j}$, where the mathematical expression for the intermolecular contact between polysaccharides ($C_{i,j}$) is included in the Methods. Analysis was performed using the Python based interface of VMD 1.9.4.

Supplementary Table 1. Average cell wall thickness of comparable-sized hyphae. Results are described as the averages and standard deviation of 75-200 measurements of 10 individual cells in each sample. n=75 for 0.5 d apo and 2 d apo samples. n=120 for 0.5 d, 1 d, 10 d CAS-treated samples and 2 d apo samples. n=200 for all other samples. Error bars are standard deviation of each reading. A source data file is provided to document each single reading. Statistical analysis was performed using a one-tailed, unpaired t-test at 99% confidence level (*, $p < 0.01$) comparing with and without drug cell walls at each time point.

Sample		0.5 d	1 d	2 d	3 d	10 d
Average cell wall thickness (nm) *	apo	24 ± 10	41 ± 10	61 ± 16	133 ± 24	123 ± 26
	+drug	84 ± 29	90 ± 28	142 ± 30	182 ± 29	185 ± 32

Supplementary Table 2. Molar composition of polysaccharides in the rigid phase. The numbers are estimated using integrals (volume) of cross peaks in 2D 53-ms CORD ^{13}C-^{13}C spectra. The average integrals of cross-peaks of each polysaccharide are shown. Error bars are standard errors of the peak integrals.

**3 d, apo**							
β-glucans		α-1,3-glucan			chitin	chitosan	
38.7%		43.9%				1.8%	
β-1,3-	β-1,4	a	b	c		a	b
35 ± 3	3.7 ± 0.9	39 ± 5	3 ± 1	1.9 ± 0.3	15 ± 2	0.8 ± 0.1	1.0 ± 0.1

**3 d, +drug**							
β-glucans		α-1,3-glucan			chitin	chitosan	
3.7%		61%				12%	
β-1,3	β-1,4	a	b	c		a	b
1.2 ± 0.2	2.5 ± 0.4	27 ± 5	21 ± 4	13 ± 4	23 ± 2	6 ± 1	6 ± 3

**10 d, apo**							
β-glucans		α-1,3-glucan			chitin	chitosan	
33%		58%					
β-1,3	β-1,4	a	b	c		a	b
16± 1	17 ± 3	36 ± 5	10.6 ± 0.7	8 ± 2	9 ± 1	2 ± 1	1.0 ± 0.1

**10 d, +drug**							
β-glucans		α-1,3-glucan			chitin	chitosan	
5%		67%					
β-1,3	β-1,4	a	b	c		a	b
0.7 ± 0.1	3.3 ± 0.4	33 ± 5	16 ± 3	16 ± 3	20 ± 2	7 ± 1	5 ± 2

The area of the following well-resolved cross peaks 53-ms CORD spectra are used:
β-1,3: the average of C1-C2/3/4/5, C2-C4, C3-C2/4/5/6, and C5-C2/4/6.
β-1,4: the average of C1-6, C2-6, C4-6, and C5-6.
α-1,3-glucan (type a): the average of C1-C2/3/5/6 and C3-2/5/6.
α -1,3-glucan (type b): the average of C1-C2 and C3-2.
α-1,3-glucan (type c): the average of C1-C3 and C3-2/4/6.
Chitin: the average of C1-2/4/5, C3/5-2, C4-2/3/5, and C6-2.
Chitosan (type a and b): the average of C1-C2 and C3-C2.

Supplementary Table 3. Chemical analysis using GC-MS. The same samples prepared using minimum medium and analyzed by ssNMR were subjected to chemical analysis. Chemical analysis was done using GC-MS coupled with enzymatic degradation. Alkali-insoluble (AI), alkali-soluble (AS), Undetected (UD).

Component	3 d, apo		3 d, +drug	
	AI	AS	AI	AS
Glucose	49.5	60	23	51
Galactose	18	22	17	28
Mannan	5.5	1	4	1
GlcNAc	23	UD	47	UD
GalNAc	4	17	9	20

Supplementary Table 4. Molar composition of mobile polysaccharides in cell walls. The numbers are estimated using integrals (volume) of spin pair peaks in 2D J-INADEQUATE ^{13}C-^{13}C spectra. The average integrals of cross-peaks of each polysaccharide are shown. Error bars are standard errors of the peak integrals. Undetected: UD.

**3 d, apo**											
β-1,3-1,6-glucans		α-1,3-glucan		Galactosaminogalactan (GAG)			Galactomannan (GM)			Chitosan	
26%		4%		48%			20%			2%	
β-1,3	β-1,3 (Br)	b	c	Gal	GalN	GalNAc	Mn^{1,2}	Mn^{1,6}	Gal*f*	a	b
24 ± 4	1.8 ± 0.4	3 ± 2	1 ± 1	33 ± 4	9 ± 1	6.2 ± 0.7	3 ± 3	2.2 ± 0.8	15 ± 2	UD	1.7 ± 0.3

**3 d, +drug**											
β-1,3-1,6-glucans		α-1,3-glucan		Galactosaminogalactan (GAG)			Galactomannan (GM)			Chitosan	
10%		33%		24%			23%			11%	
β-1,3	β-1, 3 (Br)	b	c	Gal	GalN	GalNAc	Mn^{1,2}	Mn^{1,6}	Gal*f*	a	b
7 ± 2	3.3 ± 0.5	12 ± 1	21 ± 2	15 ± 3	5 ± 1	3.4 ± 0.3	7 ± 2	5.6 ± 0.4	10 ± 1	6 ± 1	5 ± 1

**10 d, apo**											
β-1,3-1,6-glucans		α-1,3-glucan		Galactosaminogalactan (GAG)			Galactomannan (GM)			Chitosan	
13%		3%		67%			15%			1%	
β-1,3	β-1, 3 (Br)	b	c	Gal	GalN	GalNAc	Mn^{1,2}	Mn^{1,6}	Gal*f*	a	b
13.3 ± 0.8	UD	1.7 ± 0.6	1 ± 1	35 ± 4	19 ± 1	13 ± 1	6.3 ± 0.3	5 ± 2	4 ± 1	0.25 ± 0.02	0.9 ± 0.1

**10 d, +drug**											
β-1,3-1,6-glucans		α-1,3-glucan		Galactosaminogalactan (GAG)			Galactomannan (GM)			Chitosan	
7%		28%		25%			35%			7%	
β-1,3	β-1, 3 (Br)	b	c	Gal	GalN	GalNAc	Mn^{1,2}	Mn^{1,6}	Gal*f*	a	b
6 ± 2	0.66 ± 0.05	11.7 ± 0.9	16 ± 5	13 ± 2	7 ± 1	4.6 ± 0.8	9 ± 1	9.3 ± 0.9	16 ± 4	5 ± 2	2.0 ± 0.6

The area of the following well-resolved cross peaks 53-ms CORD spectra are used: the average of C1 and C2 spin pair for β-1,3, β-1,3 (Br), α-1,3 (b and c), Gal, GalN, GalNAc, Mn^{1,2}, and Mn^{1,6}, the average of C4 and C3 spin pair for Gal*f*.

Supplementary Table 5. Glucan comparison between chemical analysis and ssNMR. Alkali-insoluble (AI), alkali-soluble (AS), the fraction by whole cell wall (F_{cw}), glucan in each fraction (F_{Glc}), β-glucan in each fraction (F_{β}), α-glucan in each fraction (F_{α}), polysaccharide percentage in whole cell wall using GC-MS ($poly_{GC\text{-}MS}$), polysaccharide percentage in rigid part of the cell wall using solid-state NMR ($poly_{ssNMR}$). F_{cw}, F_{Glc}, F_{β}, and F_{α} obtained by GC-MS and enzymatic degradation.

Sample	F_{cw}		F_{Glc}	F_{β}	F_{α}	$poly_{GC\text{-}MS}$		$poly_{ssNMR}$	
						α-1,3	β-1,3/1,4	α-1,3	β-1,3/1,4
3 d, apo	AI	50.73	49.5	94.44	5.56	54	46	53	47
	AS	49.27	60	5.25	94.75				
3 d, +drug	AI	42.05	22	83.71	16.29	79	21	94	6
	AS	57.95	51	1.15	98.85				

Supplementary Table 6. Relative ratios between polysaccharides, proteins, and lipids. The ratios denote the fractions of carbons distributed in the three types of molecules, which are quantified using the areas of their corresponding spectral regions in the 1D spectra. The lipid/protein-to-polysaccharide ratios were similar in the rigid phase but changed in the mobile domains.

A. fumigatus with caspofungin (3 d and 10 d)						
Experiment	Dynamics	Component	3 d, apo	3 d, +CAS	10 d, apo	10 d, +CAS
1D CP	Rigid	Polysaccharides	0.87	0.87	0.88	0.85
		Lipids + proteins	0.13	0.13	0.12	0.15
Difference (30s - 2s)		Polysaccharides	0.86	0.87	0.88	0.87
		Lipids + proteins	0.14	0.13	0.12	0.13
1D DP (2 s)	Mobile	Polysaccharides	0.64	0.71	0.77	0.74
		Lipids + proteins	0.36	0.29	0.23	0.26
1D DP (30 s)	All	Polysaccharides	0.70	0.75	0.80	0.78
		Lipids + proteins	0.30	0.25	0.20	0.22
A. fumigatus with caspofungin (0.5, 1, and 2 d)						
Experiment	Dynamics	Component	0.5 d, +CAS	1 d, +CAS	2 d, +CAS	
1D CP	Rigid	Polysaccharides	0.62	0.87	0.72	
		Lipids + proteins	0.38	0.13	0.28	
1D DP (2 s)	Mobile	Polysaccharides	0.82	0.68	0.66	
		Lipids + proteins	0.18	0.32	0.34	
1D DP (30 s)	All	Polysaccharides	0.87	0.72	0.68	
		Lipids + proteins	0.13	0.28	0.32	
A. fumigatus with anidulafungin or micafungin (3 d)						
Experiment	Dynamics	Component	3 d, +anidulafungin		3 d, +micafungin	
1D CP	Rigid	Polysaccharides	0.77		0.81	
		Lipids + proteins	0.23		0.19	
1D DP (2 s)	Mobile	Polysaccharides	0.75		0.70	
		Lipids + proteins	0.25		0.30	
1D DP (35 s)	All	Polysaccharides	0.77		0.73	
		Lipids + proteins	0.23		0.27	

The integral of the following areas in 1D CP, 1D DP (2 s), and 1D DP (30-35 s) spectra are used: 52-92 ppm and 98-108 ppm for polysaccharides; 2-52 ppm for lipids and proteins.

Supplementary Table 7. Water-edited intensities of polysaccharide carbon sites. The intensity ratios are obtained by comparing the peak intensities in water-edited and control 2D spectra. The average values for each molecule in each sample are highlighted in bold. Error bars are standard deviations propagated from NMR signal-to-noise ratios. The error margin is typically below 10% of the reported values. Only those error bars above 10% of the reported values are shown.

Cross peaks	3 d, apo	3 d, drug	Cross peaks	3 d, apo	3 d, drug
**Average**	**0.68**	**0.38**	**Average**	**0.37**	**0.27**
B1-2	0.63	0.31	A^{a}1-2/5	0.36	0.30
B1-3	0.78	0.6±0.5	A^{a}1-3	0.34	0.28
B1-4	0.74	0.47±0.04	A^{a}1-4	0.41	0.33
B1-5	0.76	-	A^{a}1-6	0.38	0.27
B1-6	0.71	0.38	A^{a}2/5-1	0.39	0.32
B2-1	0.63	0.31	A^{a}2/5-3	0.37	0.27
B2-3	0.76	-	A^{a}2/5-4	0.43	-
B2-4	0.74	0.50	A^{a}2/5-6	0.39	0.31
B2-5	0.66	0.26	A^{a}3-1	0.33	0.27
B2-6	0.63	0.28	A^{a}3-2/5	0.35	0.27
B3-1	0.80	-	A^{a}3-4	0.42	0.24
B3-2	0.78	0.4±0.3	A^{a}3-6	0.37	0.30
B3-4	0.71	0.45	A^{a}4-1	0.36	0.25
B3-5	0.84	-	A^{a}4-2/5	0.35	0.21
B3-6	0.66	0.22±0.08	A^{a}4-3	0.35	0.23
B4-1	0.73	-	A^{a}4-6	0.39	0.24
B4-2	0.63	0.49±0.02	**Average**	**0.47**	**0.34**
B4-3	0.60	-	A^{b}1-2	0.46	0.28
B4-5	0.62	0.49±0.02	A^{b}3-2	0.57	0.33
B4-6	0.48	0.28	A^{b}2-1	0.42	0.42
B5-1	0.68	0.5±0.1	A^{b}2-3	0.45	0.32
B5-2	0.57	0.26	**Average**	**0.33**	**0.30**
B5-3	0.74	-	A^{c}1-3	0.43	0.31
B5-4	0.58	0.35	A^{c}3-1	0.2±0.1	0.34
B5-6	0.64	0.38	A^{c}3-4	0.28	0.29
**Average**	**0.41**	**0.25**	A^{c}3-2	0.24	0.28
Ch1-2	0.43	0.23	A^{c}4-3	0.38	0.28
Ch1-3	0.57	0.23	A^{c}2-3	0.42	0.31
Ch1-4	0.43	0.20	**Average**	**0.81**	**0.46**
Ch1-5	0.40	0.24	G4-6	0.9±0.2	-
Ch1-6	0.71	0.38	G1-6	0.75	0.5±0.3
Ch2-1	0.34	0.20	G2-6	-	0.38
Ch2-3	0.33	0.25			
Ch2-4	0.28±0.03	0.31			
Ch2-5	0.20	0.29			
Ch2-6	0.36±0.04	0.26			
Ch3-1	0.49	0.24			
Ch3-2	0.32	0.25			
Ch3-4	0.41	0.35			
Ch3-5	0.43	0.23			
Ch3-6	0.43	0.32			
Ch4-1	0.44	0.26			
Ch4-2	0.36	0.18			
Ch4-3	0.40	0.28			
Ch4-5	0.35	0.16			
Ch4-6	0.42	0.26			
Ch5-1	0.61	0.29			
Ch5-2	0.30	0.21			
Ch5-3	0.32	0.25			
Ch5-4	0.35	0.23			
Ch5-6	0.52	0.24			

Supplementary Table 8. ^{13}C-T_1 relaxation times of polysaccharides in *A. fumigatus* cell walls. Data are shown for the 3-day-old sample, with and without treatment by caspofungin. The average values for each molecule in each sample are highlighted in bold. The data were measured using 2D ^{13}C-^{13}C correlation experiments. The data are fit using single exponential equations: $I(t) = e^{-t/T_1}$. Error bars are standard deviations of the fit parameters.

Cross peaks	3 d apo	3 d drug	Cross peaks	3 d apo	3 d drug
**Average**	**1.4**	**2.0**	**Average**	**4.4**	**3.7**
B1-2	1.7±0.2	1.7±0.5	A^{a}1-2/5	4.0±0.3	3.6±0.3
B1-3	1.06±0.07	1.9±0.5	A^{a}1-3	5.4±0.3	4.6±0.2
B1-4	2.8±0.5	3.2±0.7	A^{a}1-4	4.7±0.2	2.9±0.2
B1-5	1.10±0.04	3.2±0.7	A^{a}2/5-1	4.5±0.3	3.1±0.7
B2-1	1.4±0.2	2.0 ±0.5	A^{a}2/5-3	5.2±0.3	3.6±0.7
B2-3	1.0±0.1	-	A^{a}2/5-4	3.4±0.2	-
B2-4	2.5±0.7	1.8±0.3	A^{a}3-1	5.3±0.2	4.9±0.7
B2-5	1.21±0.06	2.0±0.6	A^{a}3-2/5	4.6±0.5	3.9±0.5
B3-1	1.40±0.09	2±1	A^{a}3-4	5.4±0.3	3.4±0.9
B3-2	1.2±0.1	1.1±0.5	A^{a}4-1	3.4±0.4	-
B3-4	2.3±0.6	-	A^{a}4-2/5	2.7±0.4	-
B3-5	1.26±0.06	-	A^{a}4-3	4.2±0.6	-
B4-1	1.8±0.3	1.2±0.1	**Average**	**0.7**	**0.4**
B4-2	1.3±0.2	1.0±0.4	A^{b}3-2	0.4±0.1	0.20±0.05
B4-3	1.2±0.2	-	A^{b}2-3	0.9±0.2	0.6±0.2
B4-5	1.27±0.03	-	**Average**	**0.7**	**0.4**
B5-1	1.06±0.03	2.8±0.5	A^{c}3-4	0.7±0.1	0.31±0.03
B5-2	0.90±0.06	2.1±0.6	A^{c}3-2	0.7±0.2	0.11±0.02
B5-3	1.1±0.1	1.5±0.2	A^{c}4-3	0.75±0.08	0.35±0.08
B5-4	1.0±0.3	1.7±0.3	A^{c}2-3	0.7±0.1	0.66±0.08
**Average**	**2.6**	**2.9**	**Average**	**5.5**	**3.0**
Ch1-2	3.7±0.3	3.6±0.3	G1-2	3.9±0.9	3±1
Ch1-3	3.0±0.1	3.5±0.5	G1-6	6±1	2.4±0.8
Ch1-4	3.6±0.1	2.8±0.6	G2-6	4.2±0.5	3.4±0.5
Ch1-5	1.8±0.2	2.9±0.6	G4-2	5.4±0.4	5.0±0.5
Ch2-1	2.4±0.2	2.7±0.6	G4-5	2.8±0.4	2.8±0.7
Ch2-3	2.6±0.5	4.2±0.7	G4-6	7.3±0.7	2.0±0.4
Ch2-4	3.0±0.3	2.1±0.8	G5-6	9±1	2.4±0.9
Ch2-5	1.8±0.3	3.3±0.5			
Ch3-1	2.5±0.1	2.0±0.7			
Ch3-2	3.6±0.5	2.5±0.6			
Ch3-4	2.0±0.4	1.9±0.5			
Ch3-5	2.2±0.3	1.8±0.6			
Ch4-1	2.6±0.5	3.8±0.9			
Ch4-2	2.5±0.4	4±1			
Ch4-3	2.8±0.6	4±1			
Ch4-5	2.3±0.7	5.4±0.8			
Ch5-1	1.8±0.1	2.0±0.6			
Ch5-2	2.3±0.5	2.2±0.6			
Ch5-3	2.5±0.6	1.1±0.6			
Ch5-4	2.7±0.7	1.8±0.7			

Supplementary Table 9. Double-exponential ^{13}C-T_1 fitting of α-glucans. Relaxation curves of type-b and -c α-1,3-gluan showed clear double exponential feature and are fit using $I(t) = Ae^{-t/T_{1A}} + Be^{-t/T_{1B}}$, where $A + B = 1$. Error bars are standard deviations of the fit parameters.

Cross peaks	Single Exponential			Double Exponential							
				3 d, apo				3 d, +CAS			
	3 d apo	3 d drug		A	^{13}C-T_1 (s)	B	^{13}C-T_1 (s)	A	^{13}C-T_1 (s)	B	^{13}C-T_1 (s)
A^b3-2	0.4±0.1	0.20±0.05		0.75	0.19±0.01	0.25	3.0±0.3	0.78	0.14±0.02	0.22	2.2±0.8
A^b2-3	0.9±0.2	0.6±0.2		0.20	0.03±0.2	0.80	1.2±0.2	0.67	0.17±0.02	0.33	2.8±0.5
A^c3-4	0.7±0.1	0.31±0.03		0.93	0.5±0.2	0.07	4.0±0.1	0.90	0.26±0.03	0.10	1.4±0.7
A^c3-2	0.7±0.2	0.11±0.02		0.71	0.20±0.03	0.29	7±2	0.55	0.02±0.01	0.45	1.0±0.2
A^c4-3	0.75±0.08	0.35±0.08		0.40	0.24±0.06	0.60	1.3±0.2	0.86	0.23±0.01	0.14	5±1
A^c2-3	0.7±0.1	0.66±0.08		0.68	0.34±0.05	0.32	3.0±0.6	0.87	0.48±0.03	0.13	6±2

Supplementary Table 10. Solid-state NMR experimental parameters for fungal cell wall characterization. T = sample temperature; B_0 = magnetic field; ν_{MAS} = MAS frequency; ns = number of scans; d_1 = recycle delay between scans; $t_{1, max}$ = maximum t_1 evolution time (for indirect dimension); $t_{1, inc}$ = increment for t_1 (for indirect dimension) evolution time; τ_{dw} = dwell time during direct FID acquisition; τ_{acq} = maximum acquisition time during direct FID detection; τ_{XY} = cross-polarization contact time during CP from channel X to channel Y; $\nu_{1H, dec}$ = dipolar decoupling field strength. DNP experiments are marked with asterisks.

Experiment	NMR Parameters															Samples
	T (K)	B_0 (T)	ν_{MAS} (kHz)	ns	d1 (s)	$t_{1, max}$ (ms)	$t_{1, inc}$ (µs)	τ_{dw} (µs)	τ_{acq} (ms)	τ_{HC} (ms)	τ_{HN} (ms)	τ_{NC} (ms)	τ_{SD} (ms)	τ_{mix} (ms)	$\nu_{1H\ dec}$ (kHz)	
1D ^{13}C CP	298	18.8	12	2048	2			7	17	1					83	3 d apo<br>3 d CAS<br>10 d apo<br>10 d CAS
1D ^{13}C DP	298	18.8	12	64-128	2 or 30			7	28						83	
2D ^{13}C-^{13}C with CORD mixing	298	18.8	12	16	1.7	7	26	7.5	18	1				53 τ_{CORD}	83	
2D ^{13}C-^{13}C DP J-INADEQUATE	298	18.8	12	8	1.5	10	20	7.5	19						83	
2D ^{15}N-^{13}C N(CA)CO with DARR mixing	298	18.8	12	64	1.7	6	100	7.5	16		0.6	5		100 τ_{DARR}	83	
2D ^{13}C-^{13}C water-edited	298	9.4	10	128-256	1.6	5	70	10	14	1			0, 4	50 τ_{PDSD}	83	
Pseudo 3D ^{13}C-T_1	298	9.4	10	80	1.6	5	110	10	16	1				50 τ_{PDSD}	83	
* 2D ^{13}C-^{13}C with PDSD mixing	92	14.1	8	4	6.7	7	36	7.5	11	0.5				100 τ_{PDSD}	71	3 d apo<br>3 d CAS
* 2D ^{13}C-^{13}C with PAR mixing	92	14.1	8	8	7.5	7	34	7.8	8	0.5				20 τ_{PAR}	71	
* 2D ^{15}N-^{13}C N(CA)CO with PDSD	92	14.1	8	32	8	3	124	7.5	13		1	4		100 or 3000 τ_{PDSD}	71	

Supplementary Table 11. Spectral deconvolution of *A. fumigatus* spectra. Deconvolution was performed 1D CP spectra using DMfit software[6] for C1 region (107-97 ppm).

Sample	polysaccharide	Chemical shifts (ppm)	Peak width (ppm)	T_2 (s)	Amplitude	Integral	%	Polymer	Total %
0.5 d, +drug	Chitin	104.18	0.73	2.17	866557.6	1.88E+08	8.88		
	Chitin	103.5	1.09	1.46	1174036	3.72E+08	17.56	Chitin	26.4
	α-1,3-glucan (a)	101.34	1.02	1.55	4045094	1.22E+09	57.61	α-1,3-glucan	73.6
	α-1,3-glucan (b)	100.35	0.79	1.99	688949.3	1.63E+08	7.69		
	α-1,3-glucan (c)	99.1	4.8	0.33	181736	1.75E+08	8.26		
1 d, +drug	Chitin	104.18	1.66	0.95	254523.9	1.17E+08	46.38		
	Chitin	103.28	0.83	1.91	116382.8	28716336	11.42	Chitin	57.8
	α-1,3-glucan (a)	101.45	1.07	1.48	307645	96668663	38.44	α-1,3-glucan	42.2
	α-1,3-glucan (b)	100.36	0.38	4.12	73969.87	8737032	3.47		
	α-1,3-glucan (c)	99.46	0.43	3.68	5667	741914.4	0.29		
2 d, +drug	Chitin	104.18	0.78	2.04	3016069	6.96E+08	11.27		
	Chitin	103.4	1.37	1.16	4183175	1.64E+09	26.5	Chitin	37.8
	α-1,3-glucan (a)	101.25	1.08	1.46	9087710	2.89E+09	46.84	α-1,3-glucan	62.2
	α-1,3-glucan (b)	100.12	0.52	3.05	2290451	3.61E+08	5.85		
	α-1,3-glucan (c)	99.04	2.85	0.56	872170.3	5.88E+08	9.53		
3 d, +drug	Chitin	103.78	1.32	2.4	968.76	182474.3	21.13		
	Chitin	103.6	1.29	2.45	136.89	25385.57	2.94	Chitin	24.1
	α-1,3-glucan (a)	101.26	1.08	2.92	3151.18	501094.9	58.02	α-1,3-glucan	64.7
	α-1,3-glucan (b)	100.07	1.24	2.56	153.91	27340.32	3.17	Chitosan	11.2
	α-1,3-glucan (c)	98.98	0.75	4.24	278	30330.64	3.51		
	Chitosan	98.5	2.95	1.07	297.5	96963.77	11.23		
10 d, +drug	Chitin	103.75	1.31	2.42	915.55	171275.3	24.12		
	Chitin	103.63	1.02	3.1	190.5	28505.64	4.01		
	α-1,3-glucan (a)	101.21	0.95	3.33	3186.88	448853.6	63.2	Chitin	28.1
	α-1,3-glucan (b)	99.98	0.43	7.35	51	3354.32	0.47	α-1,3-glucan	68.7
	α-1,3-glucan (c)	98.97	0.65	4.88	368.89	35337.06	4.98	Chitosan	3.2
	Chitosan	97.33	0.83	3.81	248.67	22884.31	3.22		

Supplementary Table 12. ^{13}C and ^{15}N chemical shifts of biomolecules in *A. fumigatus* cell walls at ambient temperature. Superscripts are used to denote different allomorphs. Not applicable (/). Unidentified (-). Branched (Br). Reducing end (O).

Carbohydrate		C1	C2	C3	C4	C5	C6	CO	CH_3	N	Experiment	References
α-1,3-glucan	a	101	71.9	84.6	69.5	71.7	60.5	/	/	/	^{13}C-^{13}C CORD	Bhanja *et al.*2014[7]
	b	99.9	71.0	81.1	73.1	71.9	60.8	/	/	/	^{13}C-^{13}C CORD, ^{13}C DP J-INADEQUATE	
	c	100.9	69.4	79.1	71.9	70.8	60.9	/	/	/		
β-1,3-glucan		103.6	74.4	86.4	68.7	77.1	61.3	/	/	/	^{13}C-^{13}C CORD	Shim *et al.* 2007[8] Fairweather *et al.* 2009[9] Saito *et al.* 1979[10]
β-1,4-glucose units		103.3	69.4	71.7	85.3	74.3	63.4	/	/	/		Kang *et al.* 2018[11]
β-1,3-glucan (B^{Br})		103.2	73.9	85.2	69.1	76.2	69.7	/	/	/	^{13}C DP J-INADEQUATE	Lowman et al. 2011[12]
chitin		103.6	55.5	72.9	83.0	75.7	60.9	174.8	22.6	123.6	^{13}C-^{13}C CORD	Kono *et al.* 2004[13]
chitosan	a	98.8	50.9	67.3	76.9	71.7	60.2	/	/	32.9	^{13}C-^{13}C CORD, ^{13}C DP J-INADEQUATE	Fernando et al. 2021[14]
	b	96.6	51.6	66.6	76.6	71.7	61.1	/	/	32.9		
$Mn^{1,2}$		101.7	79.1	71.3	68.3	74.3	62.3	/	/	/	^{13}C DP J-INADEQUATE	Latgé *et al.*1994[15] Chakraborty *et al.* 2021[16]
$Mn\text{-}O^{1,2}$		99.4	79.1	71.3	68.3	74.3	62.3	/	/	/		
$Mn^{1,6}$		101	72.9	73.8	67.9	72.8	66.6	/	/	/		
Gal*f*		107.5	81.6	77.7	83.5	71.5	63.5	/	/	/		
Gal		93.2	72.2	70.7	73.5	72.5	60.9	/	/	/		Fontaine *et al.* 2011[17]
GalN		91.7	54.8	71.1	76.9	-	-	/	-	-		
GalNAc		95.7	57.5	75.2	81.1	-	-	175.2	22.7	-		

Amino Acids	Cα	Cβ	Cγ/γ1	Cγ2	Cδ/δ1	Amino Acids	Cα	Cβ	Cγ/γ1	Cγ2	Cδ/δ1	References
Glutamic Acid (E)	55.2	27.2	33.8			Valine (V)		29.2	18.4			Fritzsching *et al.* 2013[12]
Methionine (M)		32.0	29.5			Leucine (L)	54.1	40.1	24.6		22.4	
Histidine (H)		27.9				Alanine (A)	51.3	16.6				
Arginine (R)		29.5	26.8		39.5	Proline (P)	61.1	29.1	24.8			
Cysteine (C)	55.0	30.5				Lysine (K)	55.1	30.9	21.5			
Isoleucine (I)		36.1		15.0								

Supplementary Table 13. Chemical shifts of polysaccharides at DNP condition. Superscripts are used to denote different allomorphs. Not applicable (/). Unidentified (-). Ambiguous (_).

Carbohydrate		C1	C2	C3	C4	C5	C6	CO	CH_3	N	Experiment	Sample
α-1,3-glucan		101.1	71.4	84.9	69.9	71.3	60.2	/	/	/	^{13}C-^{13}C PAR and PDSD	3 d, apo
β-1,3-glucan		103	74.1	84.6	68.9	77.8	61.7	/	/	/		
β-1,4-glucose units		102.9	69.0	72.6	84.6	74.0	63.1	/	/	/		
chitin	a	102.7	55.6	73.0	81.2	75.1	58.3	176.6	22.4			
	b	103.0	55.6	72.9	82.0	73.6	58.8	175.3	22.7			
	c	103.1	55.0	72.5	81.3	75.0	59.3	173.8	22.7			
	d	102.2	55.9	73.1	82.0	72.7	59.1	172.6	22.5			
	e	103.2	55.2	72.5	81.1	74.2	59.0	171.7	22.6			
chitosan	a	98	51.9	67.3	76.9	73.1/69.8	57.3	/	/	33.2	^{15}N-^{13}C N(CA)CX with 100 ms DARR	
	b	96.8		65.7				/	/	33.2		
α-1,3-glucan		101.4	70.1	84.1	69.7	71.2	60.4	/	/		^{13}C-^{13}C PAR and PDSD	3 d, + drug
β-1,4-glucose units		102.9	69.2	72.6	84.6	74.0	63.1	/	/			
chitin	a	102.3	54.9	72.7	81.6	72.7	58.3	176.6	22.3			
	b	102.8	55.0	72.5	81.6	73.7	58.4	175.3	22.5			
	c	102.5	55.1	72.5	81.3	73.6	59.3	173.0	22.0			
	d	102.6	55.2	72.8	81.6	73.7	59.7	171.3	21.2			
chitosan	a	98.8	51.6	67.1	76.4	73.5/69.8	57.8	/	/	33.0	^{15}N-^{13}C N(CA)CX with 100 ms DARR	
	b	96.1		65.7				/	/	33.0		

Supplementary Table 14. Intermolecular interactions in 3-day-old *Aspergillus* cell walls. DNP-enhanced 20-ms PAR and ^{15}N-^{13}C N(CA)CX spectra with 3 s PDSD mixing are used to determine the intermolecular interactions. Cross peaks observed in 0.1 s PDSD and NCACX with 0.1 s PDSD mixing represent strong correlations. Mixed intermolecular interactions are underlined.

3d, apo

Cross peak	ω1 (ppm)	ω2 (ppm)	0.1 s PDSD	20 ms PAR	NCACX (0.1 s)	NCACX (3 s)
α-1,3-glucan-chitin						
ChMe-A1	22.4	101.0		x		
ChMe-A2,5	21.8	71.3		x		
ChMe-A6	22.6	60.3		x		
A2,5-ChCO	70.8	175.1		x		
A1-ChCO	101.3	174.4		x		
Ch4-A1	81.5	101.2	x	x		
ChN_H-A1	126.9	100.7				x
Ch1-A2,5	103.3	71.0	x	x		
A6-ChMe	60.3	22.4		x		
A2,5-ChMe	70.3	22.5	x	x		
A1-ChMe	100.9	22.7		x		
A1-Ch4	101.1	82.0	x	x		
A3-ChMe	84.1	22.5		x		
A1-Ch1	100.9	103.2		x		
Ch4-A3	81.5	84.4		x		
A2,5-Ch1	70.8	103.1	x	x		
ChMe-A3	22.5	84.5		x		
ChN_H-A3	124.4	84.2			x	x
A3-Ch4	83.4	81.1		x		
Ch1-A1	102.9	100.9		x		
Ch4’-A3	81.1	83.8		x		
α-1,3-glucan-chitosan						
Cs1-A1	97.3	101.7		x		
CsN-A1	33.0	100.1				x
A1-Cs1	101.3	97.2	x	x		
α-1,3-glucan-β-1,3-glucan						
B5-A1	78.1	101.1		x		
A1-B5	100.7	78.3		x		
A1-B6	100.9	62.1	x	x		
A1-B1	100.9	103.2		x		
A2,5-B1	70.8	103.1	x	x		
B1-A1	102.9	100.9		x		
β-1,3-glucan-chitin						

3d, +drug

Cross peak	ω1 (ppm)	ω2 (ppm)	0.1 s PDSD	20 ms PAR	NCACX (0.1 s)	NCACX (3 s)
α-1,3-glucan-chitin						
ChMe-A1	22.1	100.7		x		
ChMe-A2,5	22.0	71.4		x		
ChMe-A6	22.0	60.9		x		
A2,5-ChCO	69.5	173.2		x		
A1-ChCO	101.0	175.2		x		
Ch4-A1	81.4	100.7		x		
ChN_H-A1	126.8	101.1				x
Ch1-A2,5	102.7	70.0		x		
A6-ChMe	60.3	22.4		x		
A2,5-ChMe	69.3	22.0	x	x		
A1-ChMe	100.7	21.9		x		
A3-ChMe	83.6	22.5		x		
A1-Ch1	100.8	102.9		x		
Ch4-A3	81.7	83.7		x		
A2,5-Ch1	69.5	102.8		x		
ChMe-A3	22.1	83.8		x		
ChN_H-A3	124.3	84.1			x	x
A3-Ch4	83.5	82.0		x		
Ch1-A1	102.8	100.7		x		
Ch1-A3	102.8	84.1		x		
A3-Ch1	84.1	102.5		x		
A6-ChCO	60.4	173.0		x		
A6-ChCO’	60.4	175.3		x		
Ch2-A3	55.6	83.7		x		
A3-Ch2	83.6	55.6	x	x		
α-1,3-glucan-chitosan						
Cs1-A1	98.2	100.9		x		
CsN-A1	32.8	101.2				x
A1-Cs1	101.0	98.3		x		
CsN-A3	33.2	84.0				x
β-1,3/1,4-glucan-chitin						
ChMe-G6	22.0	62.1		x		
Ch6-G6	58.1	62.8		x		

ChMe-B4	22.1	68.8	x	x		
ChMe-B6	22.5	62.1		x		
B6-ChCO	61.8	175.3		x		
B6-ChCO’	62.0	173.5		x		
B5-Ch4	77.6	80.6		x		
B4-Ch4	68.4	81.7		x		
B6-Ch4	61.3	81.6		x		
Ch6-B5	58.9	78.2		x		
Ch6-B6	58.8	62.2		x		
B6-ChMe	61.9	22.5	x	x		
B4-ChMe	67.7	22.9		x		
B6-Ch6	62.2	59.0		x		
B5-Ch6	78.1	59.0		x		
Ch4-B6	81.7	62.2		x		
Ch4-B4	81.8	68.2		x		
Ch4-B5	80.8	77.7		x		
B3-ChMe	84.1	22.5		x		
B3-Ch4	84.4	82.5		x		
ChMe-B3	22.5	84.5		x		
Ch4-B3	81.5	84.4		x		
Ch4’-B3	81.1	83.8		x		
ChN_H-B3	124.4	84.2			x	x
chitin-chitin’/chitosan						
ChMe-Me’	22.9	21.4		x		
Ch2-2’	54.6	55.8		x		
Ch2’-2	55.8	54.7		x		
ChMe’-Me	21.4	22.9		x		
CsN-Ch4	32.3	81.2				x
CsN-Ch1	33.4	104.0				x
β-1,3-glucan-chitosan						
CsN-B1	33.4	104.0				x

ChN_H-G4	124.9	85.6		x
G6- Ch6	62.4	58.3	x	
G6-ChMe	58.1	62.8	x	
chitin-chitin’/chitosan				
ChMe-Me’	22.9	21.2	x	
Ch2-2’	54.3	55.7	x	
Ch2’-2	55.5	54.3	x	
ChMe’-Me	21.2	22.9	x	
ChCO-CO’	20.9	176.4	x	
ChCO’-CO	22.4	170.5	x	
CsN-Ch4	33.5	81.5		x
CsN-Ch1	31.7	102.2		x
Cs1-Ch1	96.5	102.2	x	
Ch1-Cs1	102.6	97.1	x	

Supplementary References

1 Chrissian, C. *et al.* Solid-state NMR spectroscopy identifies three classes of lipids in Cryptococcus neoformans melanized cell walls and whole fungal cells. *J. Biol. Chem.* **295**, 15083-15096 (2020).

2 Lamon, G. *et al.* Solid-state NMR molecular snapshots of Aspergillus fumigatus cell wall architecture during a conidial morphotype transition. *Proc. Natl. Acad. Sci. USA* **120**, e2212003120 (2023).

3 Fernando, L. D. *et al.* Structural adaptation of fungal cell wall in hypersaline environment. *Nat. Commun.* **14**, 7082 (2023).

4 Suttiarporn, P. *et al.* Structures of Phytosterols and Triterpenoids with Potential Anti-Cancer Activity in Bran of Black Non-Glutinous Rice. *Nutrients* **7**, 1672-1687 (2015).

5 Tuckey, R. C. *et al.* Lumisterol is metabolized by CYP11A1: discovery of a new pathway. *Int. J. Biochem. Cell. Biol.* **55**, 24-34 (2015).

6 Massiot, D. *et al.* Modelling one and two-dimensional solid-state NMR spectra. *Magn. Reson. Chem.* **40**, 70-76 (2002).

7 Bhanja, S. K. *et al.* Water-insoluble glucans from the edible fungus Ramaria botrytis. *Bioact. Carbohydr. Diet. Fibre* **3**, 52-58 (2014).

8 Shim, J. H. *et al.* Antitumor Effect of Soluble b-1, 3-Glucan from Agrobacterium sp. R259 KCTC 1019. *J. Microbiol. Biotechnol.* **17**, 1513-1520 (2007).

9 Fairweather, J. K., Him, J. L. K., Heux, L., Driguez, H. & Bulone, V. Structural characterization by ^{13}C-NMR spectroscopy of products synthesized in vitro by polysaccharide synthases using ^{13}C-enriched glycosyl donors: application to a UDP-glucose:(1→ 3)-β-D-glucan synthase from blackberry (Rubus fruticosus). *Glycobiology* **14**, 775-781 (2004).

10 Saitô, H., Ohki, T. & Sasaki, T. A 13C-nuclear magnetic resonance study of polysaccharide gels. Molecular architecture in the gels consisting of fungal, branched (1→ 3)-β-D-glucans (lentinan and schizophyllan) as manifested by conformational changes induced by sodium hydroxide. *Carbohydr. Res.* **74**, 227-240 (1979).

11 Kang, X. *et al.* Molecular architecture of fungal cell walls revealed by solid-state NMR. *Nat. Commun.* **9**, 1-12 (2018).

12 Lowman, D. W. *et al.* New Insights into the Structure of (1→3,1→6)-β-D-Glucan Side Chains in the Candida glabrata Cell Wall. *PLoS One* **6**, e27614 (2011).

13 Kono, H., Numata, Y., Erata, T. & Takai, M. ^{13}C and ^{1}H resonance assignment of mercerized cellulose II by two-dimensional MAS NMR spectroscopies. *Macromolecules* **37**, 5310-5316 (2004).

14 Fernando, L. D. *et al.* Structural polymorphism of chitin and chitosan in fungal cell walls from solid-state NMR and principal component analysis. *Front. Mol. Biosci.*, 727053 (2021).

15 Latge, J. P. *et al.* Chemical and immunological characterization of the extracellular galactomannan of Aspergillus fumigatus. *Infect. Immun.* **62**, 5424-5433 (1994).

16 Chakraborty, A. *et al.* A molecular vision of fungal cell wall organization by functional genomics and solid-state NMR. *Nat. Commun.* **12**, 1-12 (2021).

17 Fontaine, T. *et al.* Galactosaminogalactan, a new immunosuppressive polysaccharide of Aspergillus fumigatus. *PLoS Pathog.* **7**, e1002372 (2011).
