# Supplementary material for: Adaptative survival of Aspergillus fumigatus to echinocandins arises from cell wall remodeling beyond β−1,3-glucan synthesis inhibition
Source: Nat Commun. 2024 Jul 31;15:6382. doi: 10.1038/s41467-024-50799-8 (PMC11291495; doi:10.1038/s41467-024-50799-8)
Supplement: Supplementary file 3 — Description of Additional Supplementary Files [file 41467_2024_50799_MOESM3_ESM.pdf]

Description of Additional Supplementary Files:

Supplementary Movie 1: Molecular simulation of the interactions of polysaccharides in A. fumigatus cell walls. The solvent box is shown as a transparent glass surface and represents the field of view (FOV). Different polysaccharides are represented by spheres colored based on the atom – carbon (gray), oxygen (red), nitrogen (blue), hydrogen (white). The polymers inside the solvent box (FOV) appear as bright spheres, and fade as they diffuse into neighboring periodic images in the movie.

Supplementary Movie 2: Interaction between chitin, chitosan and α-1,3-glucan observed in a microsecond-long molecular simulation. The solvent box is shown as a transparent glass surface and the FOV. The polymers are represented by spheres and colored based on the atom type – carbon (gray), oxygen (red), nitrogen (blue), hydrogen (white). The polymers inside the solvent box (FOV) appear as bright spheres, and fade as they diffuse into neighboring periodic images in the movie. During the course of the simulation, the polysaccharides make both short (<5Å) and long-range (<10Å) interactions with each other.

Supplementary Movie 3: Interaction between chitin, chitosan and β-glucan polymers observed in a microsecond-long molecular simulation. The solvent box is shown as a transparent glass surface and the FOV. The polymers are represented by spheres and colored based on the atom – carbon (gray), oxygen (red), nitrogen (blue), hydrogen (white). The polymers inside the solvent box (FOV) appear as bright spheres, and fade as they diffuse into neighboring periodic images in the movie. During the course of the simulation, the polysaccharides make both short (<5Å) and long-range (<10Å) interactions with each other.

Supplementary Movie 4: Interaction between chitin and chitosan polymers observed in a microsecond long molecular simulation. The solvent box is shown as a transparent glass surface and the FOV. The polymers are represented by spheres and colored based on the atom – carbon (gray), oxygen (red), nitrogen (blue), hydrogen (white). The polymers inside the solvent box (FOV) appear as bright spheres, and fade as they diffuse into neighboring periodic images in the movie. The two chitin polymers maintain a stacked conformation, while both chitosan and chitinchitosan copolymers make short (<5Å) and long-range (<10Å) interactions with each other.

Supplementary Movie 5: Interaction between two chitin polymers observed in a microsecond long molecular simulation. The solvent box is shown as a transparent glass surface and the FOV. The two chitin polymers are represented by spheres and colored based on the atom – carbon (gray), oxygen (red), nitrogen (blue), hydrogen (white). The polymers in the solvent box (FOV) appear as bright spheres, and fade as they diffuse into neighboring periodic images in the movie. We observe that the two chitin polymers interact to form a stacked conformation which is stable over the length of the simulation.
