# Supplementary material for: Adaptative survival of Aspergillus fumigatus to echinocandins arises from cell wall remodeling beyond β−1,3-glucan synthesis inhibition
Source: Nat Commun. 2024 Jul 31;15:6382. doi: 10.1038/s41467-024-50799-8 (PMC11291495; doi:10.1038/s41467-024-50799-8)
Supplement: Supplementary file 9 — Reporting Summary [file 41467_2024_50799_MOESM9_ESM.pdf]

nature portfolio

Corresponding author(s): Tuo Wang
Last updated by author(s): 07/10/2024

Reporting Summary

Nature Portfolio wishes to improve the reproducibility of the work that we publish. This form provides structure for consistency and transparency in reporting. For further information on Nature Portfolio policies, see our Editorial Policies and the Editorial Policy Checklist.

Statistics

For all statistical analyses, confirm that the following items are present in the figure legend, table legend, main text, or Methods section.

n/a	Confirmed	
☐	☒	The exact sample size (*n*) for each experimental group/condition, given as a discrete number and unit of measurement
☐	☒	A statement on whether measurements were taken from distinct samples or whether the same sample was measured repeatedly
☐	☒	The statistical test(s) used AND whether they are one- or two-sided<br>*Only common tests should be described solely by name; describe more complex techniques in the Methods section.*
☒	☐	A description of all covariates tested
☒	☐	A description of any assumptions or corrections, such as tests of normality and adjustment for multiple comparisons
☐	☒	A full description of the statistical parameters including central tendency (e.g. means) or other basic estimates (e.g. regression coefficient) AND variation (e.g. standard deviation) or associated estimates of uncertainty (e.g. confidence intervals)
☐	☒	For null hypothesis testing, the test statistic (e.g. *F*, *t*, *r*) with confidence intervals, effect sizes, degrees of freedom and *P* value noted<br>*Give P values as exact values whenever suitable.*
☒	☐	For Bayesian analysis, information on the choice of priors and Markov chain Monte Carlo settings
☒	☐	For hierarchical and complex designs, identification of the appropriate level for tests and full reporting of outcomes
☒	☐	Estimates of effect sizes (e.g. Cohen's *d*, Pearson's *r*), indicating how they were calculated

Our web collection on statistics for biologists contains articles on many of the points above.

Software and code

Policy information about availability of computer code

Data collection	The data collected on Topspin version 3.5 and 2.5 and OpenVNMRJ 2.1a and TEM images Samples were observed with a JEOL JEM-1400 TEM (Peabody, MA) with an accelerating voltage of 120 kV at varying magnifications and photographed with Gatan Orius SC 1000A camera. AFM data was collected using Bruker FastScan AFM system using ScanAsystTM imaging mode.
Data analysis	The graphs were generated through OriginPro. 2019b and Origin 2021b.<br>The NMR spectra were analyzed and processed using TopSpin 4.0.8<br>Statistical analysis for TEM imaging was conducted in Microsoft Excel 365.<br>The cell wall thickness from TEM images were obtained from ImageJ V1.8.0_172<br>The figures and illustrations are drawn in Adobe Illustrator Cs6 V16.0.0<br>The hydration maps were generated using Python 3.8.4<br>AFM images were analyzed using Gwyddion software<br>Spectral deconvolution was conducted using DMfit software version dmfit#20230120<br>MD simulations were run using NAMD 2.14, NAMD 3.0a9, VMD 1.9.4a55, CHARMM-GUI Glycan Modeler, Python 3.10.12, Numpy 1.24.2, Tcl 8.6

For manuscripts utilizing custom algorithms or software that are central to the research but not yet described in published literature, software must be made available to editors and reviewers. We strongly encourage code deposition in a community repository (e.g. GitHub). See the Nature Portfolio guidelines for submitting code & software for further information.

Data

Policy information about availability of data

All manuscripts must include a data availability statement. This statement should provide the following information, where applicable:

- Accession codes, unique identifiers, or web links for publicly available datasets
- A description of any restrictions on data availability
- For clinical datasets or third party data, please ensure that the statement adheres to our policy

All NMR spectra and biochemical data that support the findings of this study are provided in the article and Supplementary Information. The topspin NMR datasets of all ssNMR spectra collected on fungal cell walls are available in the public repository Zenodo under the DOI number: https://doi.org/10.5281/zenodo.12700137. Source data are provided with this paper. All MD input data, which includes topology, force field parameters and input scripts that support the findings of this research are publicly available via Zenodo under the DOI number https://doi.org/10.5281/zenodo.12700137

Research involving human participants, their data, or biological material

Policy information about studies with human participants or human data. See also policy information about sex, gender (identity/presentation), and sexual orientation and race, ethnicity and racism.

Reporting on sex and gender	N/A
Reporting on race, ethnicity, or other socially relevant groupings	N/A
Population characteristics	N/A
Recruitment	N/A
Ethics oversight	N/A

Note that full information on the approval of the study protocol must also be provided in the manuscript.

Field-specific reporting

Please select the one below that is the best fit for your research. If you are not sure, read the appropriate sections before making your selection.

- [x] Life sciences
- [ ] Behavioural & social sciences
- [ ] Ecological, evolutionary & environmental sciences

For a reference copy of the document with all sections, see nature.com/documents/nr-reporting-summary-flat.pdf

Life sciences study design

All studies must disclose on these points even when the disclosure is negative.

Sample size	Each NMR sample contains 30-100 mg (hydrated mass) of fungal materials, which is determined by weighing the material before and after packing into a NMR rotor. The NMR spectra report the average feature of all cells in each sample. A large number of scans were collected for averaging and for each NMR spectrum, resulting in reproducibility of each spectrum. The exact number of scans are summarized in Supplementary Table 10. Three replicates are tested for with and without drug, confirming the reproducibility (Supplementary Fig. 1).
Data exclusions	There were no data exclusions.
Replication	Each NMR experiment were average by number of scans. All the parameters for NMR experiments are provided in Supplementary Information. We have performed replication 3 batches from each with and without drug at different time periods (2019-2021), each showing similar NMR fingerprints across the batches (Supplementary Fig. 1). All attempts at replication were successful.
Randomization	Different fungal samples were studied separately.
Blinding	Not Applicable to NMR studies of the fundamental structure of carbohydrate molecules. As the samples studied are natural mixtures of molecules, there is no differentiation and there is no need for randomized controlled trial.

Reporting for specific materials, systems and methods

We require information from authors about some types of materials, experimental systems and methods used in many studies. Here, indicate whether each material, system or method listed is relevant to your study. If you are not sure if a list item applies to your research, read the appropriate section before selecting a response.

Materials & experimental systems

n/a	Involved in the study
☒	☐ Antibodies
☒	☐ Eukaryotic cell lines
☒	☐ Palaeontology and archaeology
☒	☐ Animals and other organisms
☒	☐ Clinical data
☒	☐ Dual use research of concern
☒	☐ Plants

Methods

n/a	Involved in the study
☒	☐ ChIP-seq
☒	☐ Flow cytometry
☒	☐ MRI-based neuroimaging
