# Supplementary material for: Adaptative survival of Aspergillus fumigatus to echinocandins arises from cell wall remodeling beyond β−1,3-glucan synthesis inhibition
Source: Nat Commun. 2024 Jul 31;15:6382. doi: 10.1038/s41467-024-50799-8 (PMC11291495; doi:10.1038/s41467-024-50799-8)
Supplement: Supplementary file 2 — Peer Review File [file 41467_2024_50799_MOESM2_ESM.pdf]

nature portfolio

# Peer Review File

## Adaptative Survival of Aspergillus fumigatus to Echinocandins Arises from Cell Wall Remodeling Beyond β-1,3-glucan Synthesis Inhibition

**Open Access** This file is licensed under a Creative Commons Attribution 4.0 International License, which permits use, sharing, adaptation, distribution and reproduction in any medium or format, as long as you give appropriate credit to the original author(s) and the source, provide a link to the Creative Commons license, and indicate if changes were made. In the cases where the authors are anonymous, such as is the case for the reports of anonymous peer reviewers, author attribution should be to 'Anonymous Referee' followed by a clear attribution to the source work. The images or other third party material in this file are included in the article's Creative Commons license, unless indicated otherwise in a credit line to the material. If material is not included in the article's Creative Commons license and your intended use is not permitted by statutory regulation or exceeds the permitted use, you will need to obtain permission directly from the copyright holder. To view a copy of this license, visit http://creativecommons.org/licenses/by/4.0/.

**Editorial Note:** This manuscript has been previously reviewed at another journal that is not operating a transparent peer review scheme. This document only contains reviewer comments and rebuttal letters for versions considered at *Nature Communications*.

Reviewer #1 (Remarks to the Author):

The authors have put a lot effort to adress my previous concerns and that of other reviewers. This MS should be accepted at this point.

Reviewer #2 (Remarks to the Author):

The revised manuscript appropriately has addressed my earlier concerns. Also, several aspects have been clarified.

I recommend publication of the revised version.

Reviewer #3 (Remarks to the Author):

The authors were highly responsive to all of the reviewers concerns, and revised manuscript is much improved and should be a valuable asset to the field.

**Responses to Reviewers Comments**

We appreciate the positive responses from all three reviewers and their support for the publication of this manuscript.
